# Accelerated DNA methylation changes in middle-aged men define sexual dimorphism in human lifespans

**DOI:** 10.1186/s13148-018-0573-1

**Published:** 2018-10-29

**Authors:** Fu-Hui Xiao, Xiao-Qiong Chen, Yong-Han He, Qing-Peng Kong

**Affiliations:** 1 State Key Laboratory of Genetic Resources and Evolution/Key Laboratory of Healthy Aging Research of Yunnan Province, Kunming Institute of Zoology, Chinese Academy of Sciences, Kunming, 650223 China; 2 Center for Excellence in Animal Evolution and Genetics, Chinese Academy of Sciences, Kunming, 650223 China; 3 Kunming Key Laboratory of Healthy Aging Study, Kunming, 650223 China; 4 KIZ/CUHK Joint Laboratory of Bioresources and Molecular Research in Common Diseases, Kunming, 650223 China

**Keywords:** DNA methylation, Middle-age, Cardiovascular disease, Sexual dimorphism, Lifespan

## Abstract

**Background:**

Accelerated age-associated DNA methylation changes in males may explain the earlier onset of age-related diseases (e.g., cardiovascular disease (CVD)) and thus contribute to sexually dimorphic morbidity and lifespan. However, the details regarding the emergence of this sex-biased methylation pattern remain unclear.

**Results:**

To address these issues, we collected publicly available peripheral blood methylation datasets detected by Illumina HumanMethylation450 BeadChip platform from four studies that contain age and gender information of samples. We analyzed peripheral blood methylation data screened from 708 subjects of European ancestry. Results revealed a significant methylation change acceleration in middle-aged males (40–50 years old), which was further supported by another cohort containing 2711 subjects with Indian ancestry. Additional analyses suggested that these sexually dimorphic methylation changes were significantly overrepresented in genes associated with CVD, which may impact the potential activation of disease expression. Furthermore, we showed that higher prevalence of drinking and smoking in the males has some contribution to the sex-based methylation patterns during aging.

**Conclusion:**

Our results indicated that the sex-biased methylation changes occurred in middle-aged men in an acceleration manner and likely contribute to the sexual dimorphism observed in human lifespan by promoting the occurrence of CVD. As drinking and smoking were also found to be associated with this accelerated methylation change in men, it is possible that male lifespan may be prolonged by improving unhealthy lifestyles at or before middle age.

**Electronic supplementary material:**

The online version of this article (10.1186/s13148-018-0573-1) contains supplementary material, which is available to authorized users.

## Introduction

It is well known that men often live shorter than women [1], which is partly explained by their higher mortality and earlier onset of some age-related diseases, especially cardiovascular disease (CVD) [2, 3]. Accumulated evidence showed that epigenetic changes in aging process are associated with lifespan and age-related diseases including CVD [4–7], indicating the roles of epigenetic mechanisms in sexually dimorphic lifespan. As the well-known epigenetic modification, DNA methylation plays an important role in the regulation of gene transcription [8]. Sexually divergent DNA methylation changes during aging have been observed in animals and humans [9, 10]. Notably, a previous study showed that diverse age-related DNA methylation changes have some associations with CVD incidence difference in crowds [11]. Especially, men do possess an accelerated methylation change during aging, which is supposed to contribute to the earlier onset of CVD and shorter lifespan [11, 12]. However, the ways or causes for this sexually dimorphic methylation change pattern remain largely unclear. In this study, we have therefore analyzed the sexual differences of methylation profiles (Illumina HumanMethylation450 BeadChip) during aging in two independent cohorts containing 708 and 2711 samples [12–15], respectively, and explored the contribution of some risk factors (i.e., drinking and smoking) to these sexual methylation differences.

## Results and discussion

**Fig. 1:**
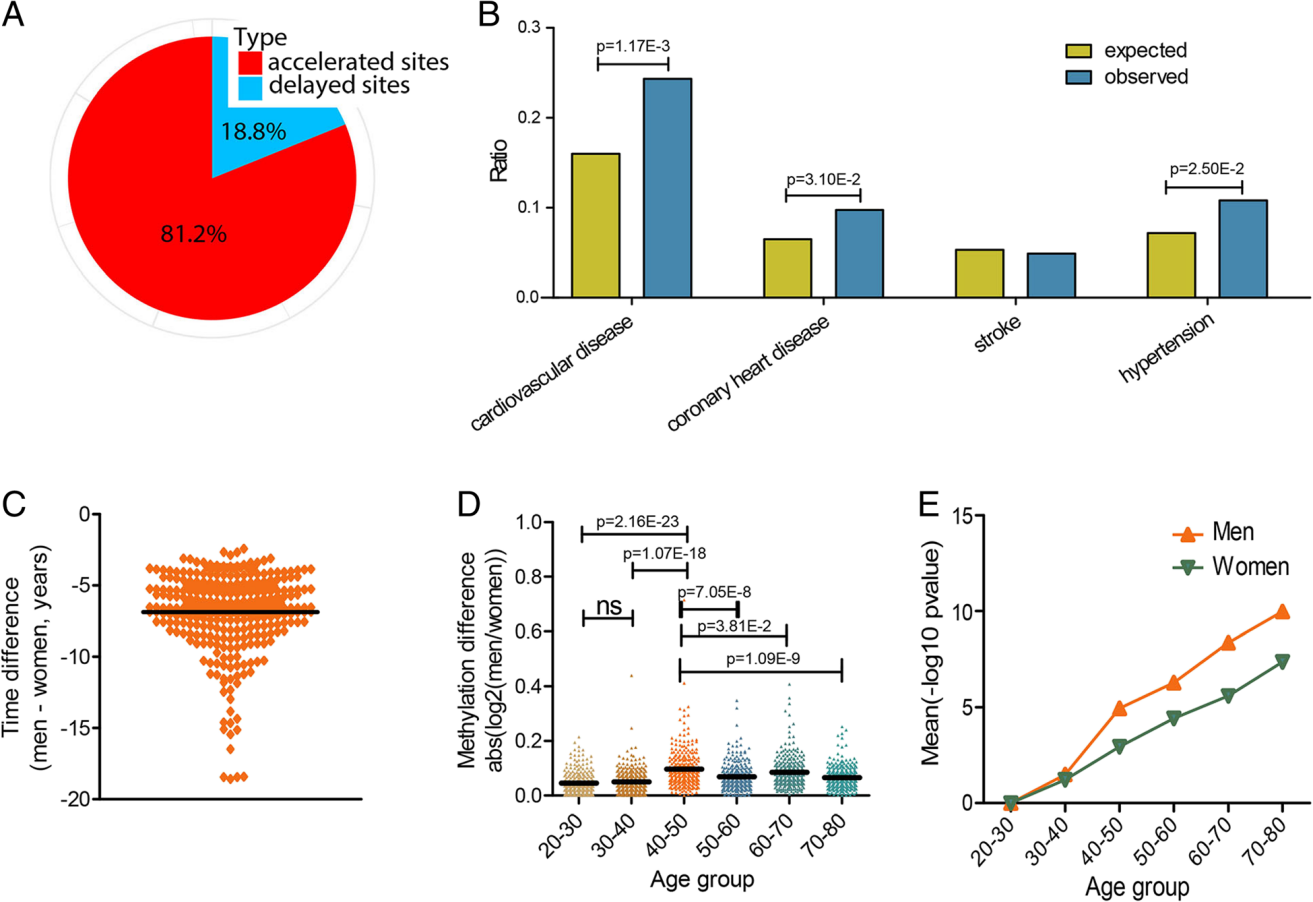
CpGs exhibiting sexually dimorphic methylation changes during aging. **a** Percentage of accelerated and delayed CpGs in men. **b** Distribution of accelerated CpGs in genes associated with CVD. **c** Time differences in accelerated CpGs between the two sexes. **d** Sex-related differences in methylation of accelerated CpGs in various age groups. **e** Methylation changes in accelerated CpGs between the older groups and youngest control group (20–30 years old) for each gender. ns non-significant

By analyzing the methylation profile data (covering over 485,000 CpG sites) from 708 healthy individuals (of European ancestry), screened by the Illumina HumanMethylation450 BeadChip (Additional file 1: Table S1) [12–14], we identified 357 CpGs showing significant sexually dimorphic changes in methylation (see the “Materials and Methods” section). Interestingly, changes in most of these CpGs (290/357) were accelerated in men (Fig. 1a and Additional file 1: Table S2). We annotated the distribution of the above 290 CpGs and found they were located on 185 genes, the overrepresentation of which are associated with CVD (Fig. 1b and Additional file 1: Table S2). For example, cg14519515 locates in the promoter of *ADRBK1*, a gene with elevated expression in CVD patients [16, 17]. The decreased methylation level of this CpG site during aging likely upregulates the expression of *ADRBK1* in men and contributes to the earlier onset of CVD (Additional file 1: Figure S1A, B). An additional case comes from cg20222376. This CpG site locates in the promoter of *AKAP8L* and displays reduced methylation level with age (Additional file 1: Figure S1C). The methylation level of cg20222376 is positively associated with the expression level of *AKAP8L* (Additional file 1: Figure S1D). Given the high expression of AKAPs can protect the cardiovascular system [18] and men contain a lower methylation level at this CpG locus compared with age-matched women, it is likely that the methylation pattern will increase the risk of CVD in men. Similar to observations reported in previous research [11], our results suggested that these sex-biased methylation changes may play a role in promoting the occurrence of CVD in men. Coincidently, we also observed that methylation changes were accelerated in males and occurred 6.9 years earlier, on average, than the changes in females (Fig. 1c), echoing the observation that CVD develops 7–10 years earlier in men [3].

We next explored how these sexually dimorphic methylation differences emerge during aging. The methylation differences in the 290 identified CpGs were compared among different age groups, as shown in Fig. 1d and Additional file 1: Figure S2A, B. Surprisingly, a significantly accelerated sex-based methylation difference occurred in the 40–50-year-old age group, in sharp contrast with the observation that the degree of difference in methylation between the two sexes was relatively small before the age of 40. Consistently, age-related analysis in each gender revealed an abruptly accelerated methylation change in middle-aged men (Fig. 1e).

**Fig. 2:**
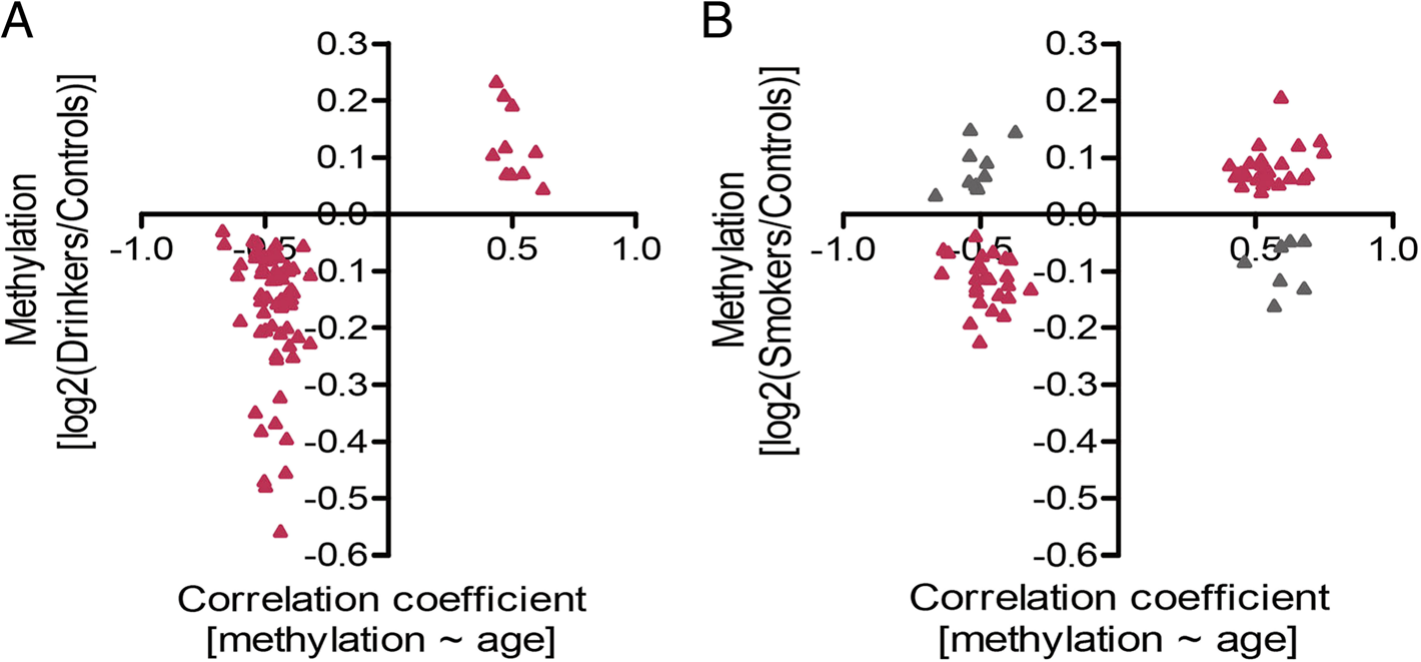
Effects of drinking and smoking on sex-related differences in methylation changes. **a** Accelerated CpGs differentially methylated in drinkers. **b** Accelerated CpGs differentially methylated in smokers

To determine the factors associated with the accelerated methylation change in middle-aged males, we analyzed the influence of drinking and smoking, two well-known risk factors with higher prevalence in men that contribute, at least in part, to their high risk of CVD and shorter lifespan [19–24]. By analyzing available public methylation dataset with drinking information [25], we found that, among the 290 identified CpGs, 70 showed methylation differences between drinkers and non-drinkers (*p* < 0.05). All of the 70 CpGs exhibited the same directional changes in drinkers as those with age (Fig. 2a). A similar pattern was observed in smokers by the methylation dataset collected in females (the males being unavailable) [26]. Sixty-seven of the 290 CpGs were differentially methylated in smokers compared with non-smokers (*p* < 0.05), with most of them CpGs (51/67) showing the same directional changes in smokers as those with age (Fig. 2b).

**Fig. 3:**
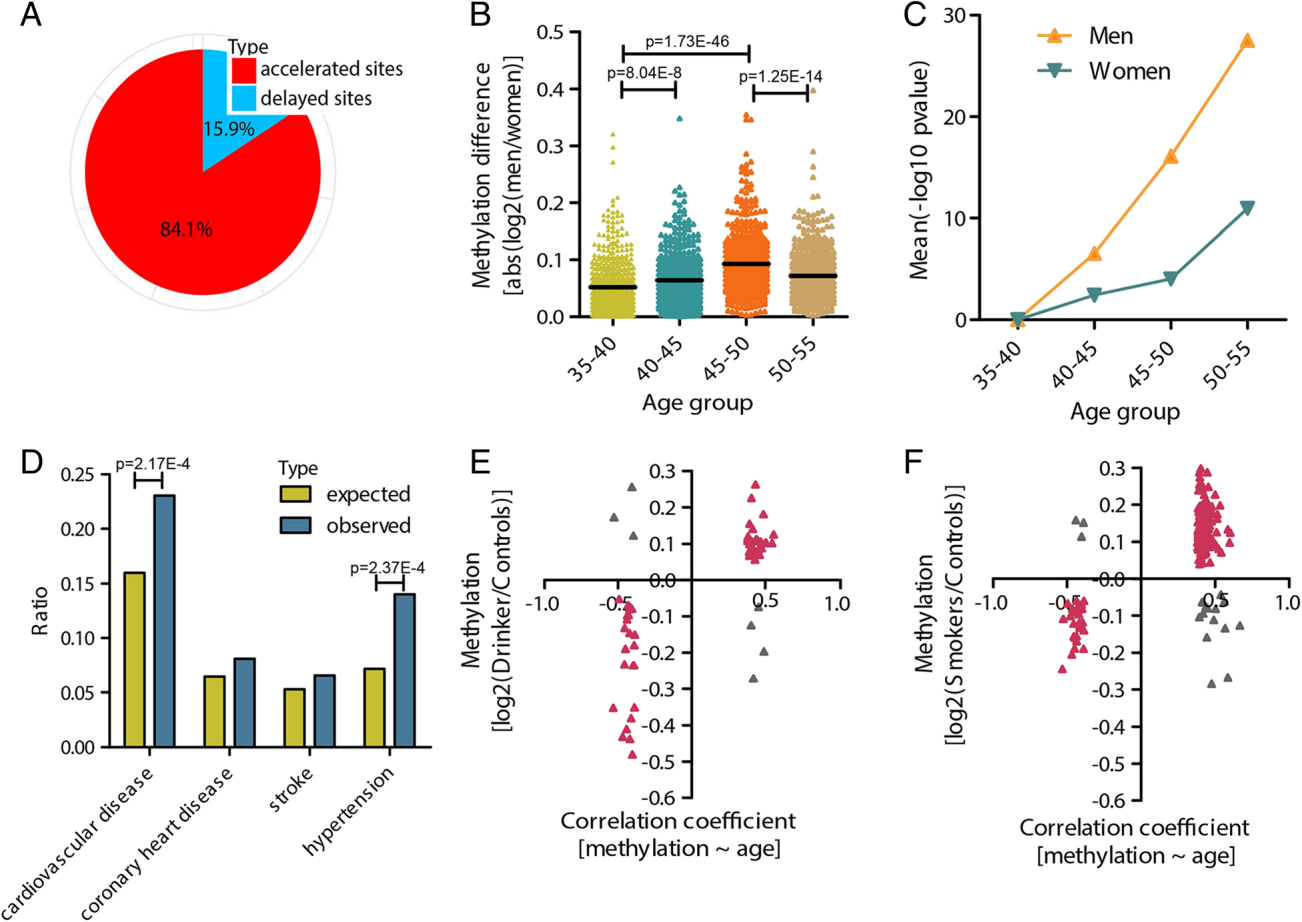
Sexually dimorphic DNA methylation patterns observed in an independent population. **a** Percentage of accelerated and delayed CpGs in men. **b** Methylation differences in accelerated CpGs in each group between the two sexes. **c** Significant degree of methylation changes in accelerated CpGs with increasing age compared to the young group (35–40 years old). **d** Distribution of accelerated CpGs in genes associated with CVD. **e** Accelerated CpGs with methylation differences between drinkers and non-drinkers. **f** Accelerated CpGs with methylation differences between smokers and non-smokers

To test whether our observations were confined to a certain population or could be observed in others, we analyzed another methylation dataset from London Life Sciences Prospective Population (LOLIPOP) study containing 2711 subjects with Indian ancestry (Additional file 1: Table S1) [15]. We identified 709 CpGs that exhibited sex-based differences in methylation during aging, with most (596/709) showing significant acceleration in men (on average, 5.4 years earlier in males than females; Fig. 3a and Additional file 1: Table S3). Again, sharply accelerated methylation changes were observed in middle-aged men (Fig. 3b, c and Additional file 1: Figure S3A, B). Importantly, the 596 CpGs were again overrepresented in genes associated with CVD (Fig. 3d and Additional file 1: Table S3). Moreover, 66 and 180 of the CpGs were differentially methylated in drinkers and smokers, respectively (*p* < 0.05), with most (59/66, 162/180) exhibiting identical directional changes as those with age (Fig. 3e, f).

A previous study has suggested that the rapid DNA methylation changes during aging in men contribute to the earlier onset of CVD and shorter lifespan [11]. Our study further identified that such acceleration emerges in middle-aged men, with drinking and smoking found to be associated with these changes, and thus likely has some contribution to the acceleration. This observation is in accordance with epidemiological findings of abruptly increased CVD mortality in men aged 45–54 years old [2]. Since we have observed the same pattern in the two cohorts with different ancestries and thus most plausibly distinct genetic backgrounds, it is then unlikely that our observation is race specific. Our study therefore raises the possibility that controlling these aberrant epigenetic modifications at or before middle age via lifestyle changes, e.g., smoking and alcohol reduction or abstinence, could help reduce the incidence of CVD in men and thus prolong their lifespans.

## Materials and methods

### Data collection

The methylation data, generated by the Illumina HumanMethylation450 BeadChip (HM450), were collected from NCBI’s Gene Expression Omnibus (GEO) datasets. One integrated dataset was downloaded with accession numbers GSE32148, GSE41169, and GSE40279, in which only data from healthy samples were considered (Additional file 1: Table S1) [12–14]; another dataset was downloaded with the accession number GSE55763 (Additional file 1: Table S1) [15]. Two additional datasets containing smoking and drinking information were downloaded with accession numbers GSE53045 and GSE57853 [25, 26]. The methylation level of each CpG site is represented by a beta value (range 0–1). The 364 normal tissue samples with RNA-seq (version 2) and methylation (HM450) data were downloaded from The Cancer Genome Atlas (TCGA) Data Portal website (https://tcga-data.nci.nih.gov/). Any sites with missing values in more than 5% of samples were discarded, and the remaining CpG sites with missing values in a few subjects were filled with the R “impute” package [27]. The values were then normalized using quantile normalization with the *normalize.quantiles* function in the R “preprocessCore” package (http://www.bioconductor.org/).

### Identification of CpGs with sexually dimorphic methylation changes

We first calculated the coefficient of variance (cv) of all CpG sites and filtered out those with values less than the interquartile range (IQR) cut-off of 0.5. A stepwise polynomial regression model (*step(lm(y~ 1 + Age + I(Age^2) + I(Age^3))), direction = “backward”*) was then used to find the best-fit model. Sites showing significantly age-related changes were identified using the *F* test between the best-fit and null models (*y~ 1*). The CpG sites with *p* values [*F* test, Benjamini and Hochberg (BH) corrected] < 0.01 and adjusted *R*^2^ > 0.25 were retained for further analysis. To improve the credibility of the above sites, a nonparametric Spearman rank correlation test was adopted, and only the sites with absolute correlation coefficient values of > 0.2 were regarded as age-related CpG sites. We then used multiple linear regression with the best-fit model to identify the CpG sites with sex-related differences. To test the heterochrony (age shift) for the sex-biased CpG sites, we defined the age-related methylation change curve of men as the reference object for each CpG site and used a nonlinear least-squares algorithm (NL2SOL) to find the optimal age shift between the two sexes. The significance of the age transformation was assessed using the *F* test, as described in detail elsewhere [28]. Here, the age  shift for each CpG was calculated, in which “age-shift < 0” represents the curve of men located on the left side of women and thus defined as “accelerated sites in men”, otherwise, on the right side.

### Gene annotation, enrichment, and correlation analyses

Gene information on the CpG sites of interest was obtained from the “IlluminaHumanMethylation450k.db” database. The genes associated with age-related diseases were collected from GeneCards version 3.12 by searching the names including cardiovascular disease, coronary heart disease, stroke, and hypertension [29]. A hypergeometric test was performed to find the enriched disease terms based on the observed and expected gene numbers. A Pearson correlation test was performed to test the relationship between the methylation of each CpG site and the expression of its corresponding gene with the data from TCGA, including the 364 normal tissue samples with both RNA-seq and HM450 data (https://tcga-data.nci.nih.gov/, see the “Data collection” section).

### Analysis of sex-related methylation change patterns

The mean methylation differences in one age group between the two sexes for each CpG were calculated and used to compare with adjacent age groups. We also evaluated the significant degree of methylation changes with increasing age for each gender by comparing the older and youngest groups (control). Statistical significance was calculated with Student’s *t* tests.

### Identification of differentially methylated CpGs in smokers or drinkers

Using the methylation datasets with drinking and smoking information (see the “Data collection” section), we analyzed the CpGs with methylation differences between the two groups (smokers/drinkers versus controls) using Student’s *t* tests.

## Additional file

Additional file 1: **Figure S1.** Illustrations for the roles of the sexually dimorphic methylation in promoting occurrence of cardiovascular disease in men. **Figure S2.** Methylation differences of the identified 290 CpGs (with accelerated methylation changes in males) between the two sexes among different age groups. **Figure S3.** Sexually dimorphic methylation in the CpGs with accelerated methylation changes in males. **Table S1.** Summary of the collected DNA methylation datasets. **Table S2.** Information on the 290 accelerated CpG sites in the first dataset. **Table S3.** Information on the 574 accelerated CpG sites in the second dataset. (DOC 1996 kb)

## Abbreviations

ADRBK1: Adrenergic beta receptor kinase 1
AKAP8L: A-kinase-anchoring protein 8-like
BH: Benjamini and Hochberg
cv: Coefficient of variance
CVD: Cardiovascular disease
HM450: Illumina HumanMethylation450 BeadChip
IQR: Interquartile range
NL2SOL: Nonlinear least-squares algorithm
