# Supplementary material for: Accelerated DNA methylation changes in middle-aged men define sexual dimorphism in human lifespans
Source: Clin Epigenetics. 2018 Oct 29;10:133. doi: 10.1186/s13148-018-0573-1 (PMC6206726; doi:10.1186/s13148-018-0573-1)
Supplement: Supplementary file 1 — Figure S1. Illustrations for the roles of the sexually dimorphic methylation in promoting occurrence of cardiovascular disease in men. Figure S2. Methylation differences of the identified 290 CpGs (with accelerated methylation changes in males) between the two sexes among different age groups. Figure S3. Sexually dimorphic methylation in the CpGs with accelerated methylation changes in males. Table S1. Summary of the collected DNA methylation datasets. Table S2. Information on the 290 accelerated CpG sites in the first dataset. Table S3. Information on the 574 accelerated CpG sites in the second dataset. (DOC 1996 kb) [file 13148_2018_573_MOESM1_ESM.doc]

**Supplemental Information for “Accelerated DNA methylation changes in middle-aged men define sexual dimorphism in human lifespans”**

**Index:**

**Figure S1. Illustrations for the roles of the sexually dimorphic methylation in promoting occurrence of cardiovascular disease in men.** (A) Sexual difference of age-associated DNA methylation for cg14519515. (B) Correlation of cg14519515 methylation and *ADRBK1* expression (*p* < 0.05). (C) Sexual difference of age-associated DNA methylation for cg20222376. (D) Correlation of cg20222376 methylation and *AKAP8L* expression (*p* < 0.05).

**Figure S2. Methylation differences of the identified 290 CpGs (with accelerated methylation changes in males) between the two sexes among different age groups.** (A) CpGs with positive associations between methylation level and age. (B) CpGs with negative associations between methylation level and age.

**Figure S3. Sexually dimorphic methylation in the CpGs with accelerated methylation changes in males.** (A) Methylation differences of the accelerated CpGs between the two sexes among different age groups. (B) Significant degree of methylation changes for the accelerated CpGs between older (e.g., 40–50 years old) and youngest (i.e., 35–40 years old) groups in each gender.

**Table S1.** Summary of the collected DNA methylation datasets.

**Table S2.** Information on the 290 accelerated CpG sites in the first dataset.

**Table S3.** Information on the 574 accelerated CpG sites in the second dataset.


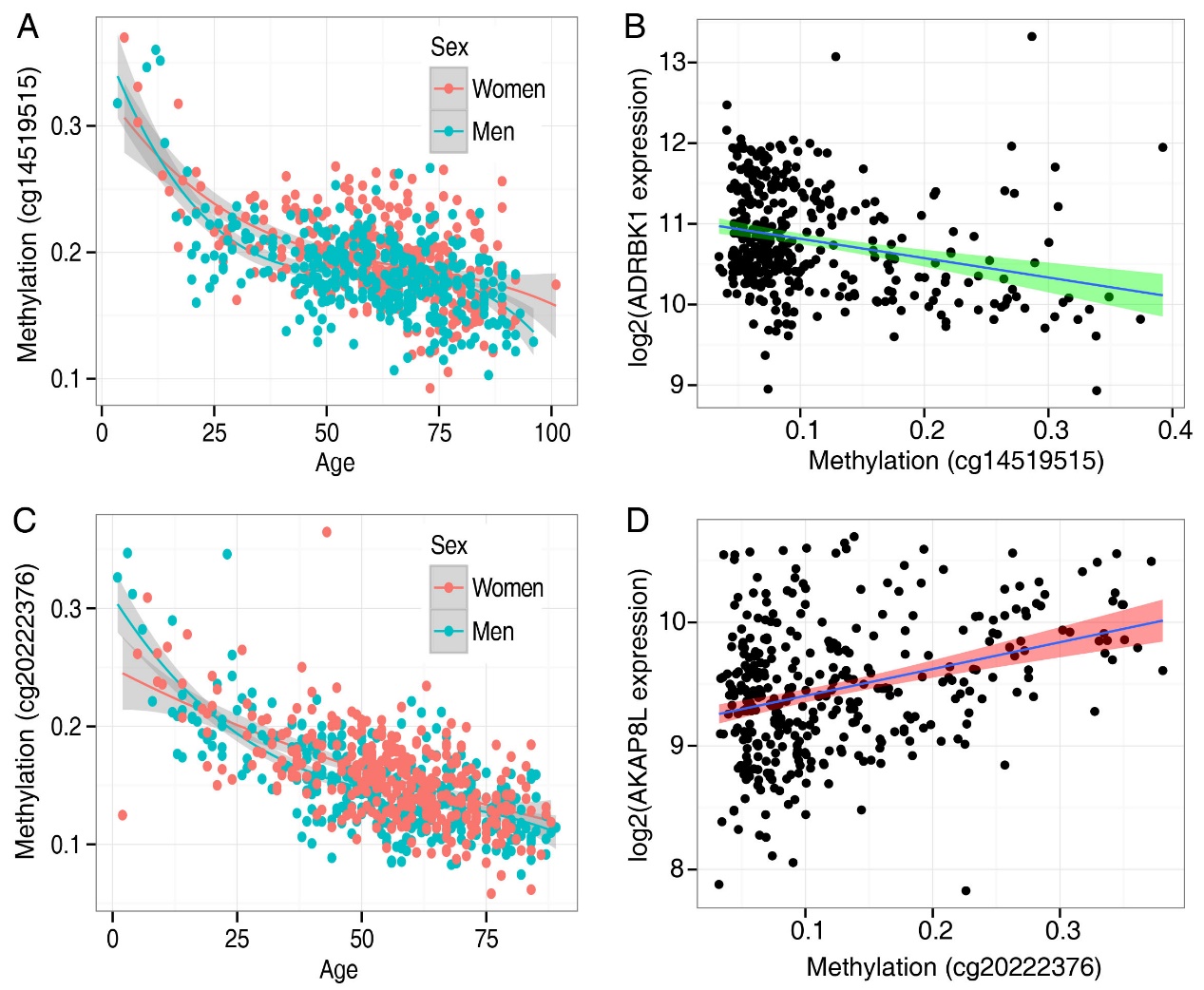


**Figure S1. Illustrations for the roles of the sexually dimorphic methylation in promoting occurrence of cardiovascular disease in men.** (A) Sexual difference of age-associated DNA methylation for cg14519515. (B) Correlation of cg14519515 methylation and *ADRBK1* expression (*p* < 0.05). (C) Sexual difference of age-associated DNA methylation for cg20222376. (D) Correlation of cg20222376 methylation and *AKAP8L* expression (*p* < 0.05).


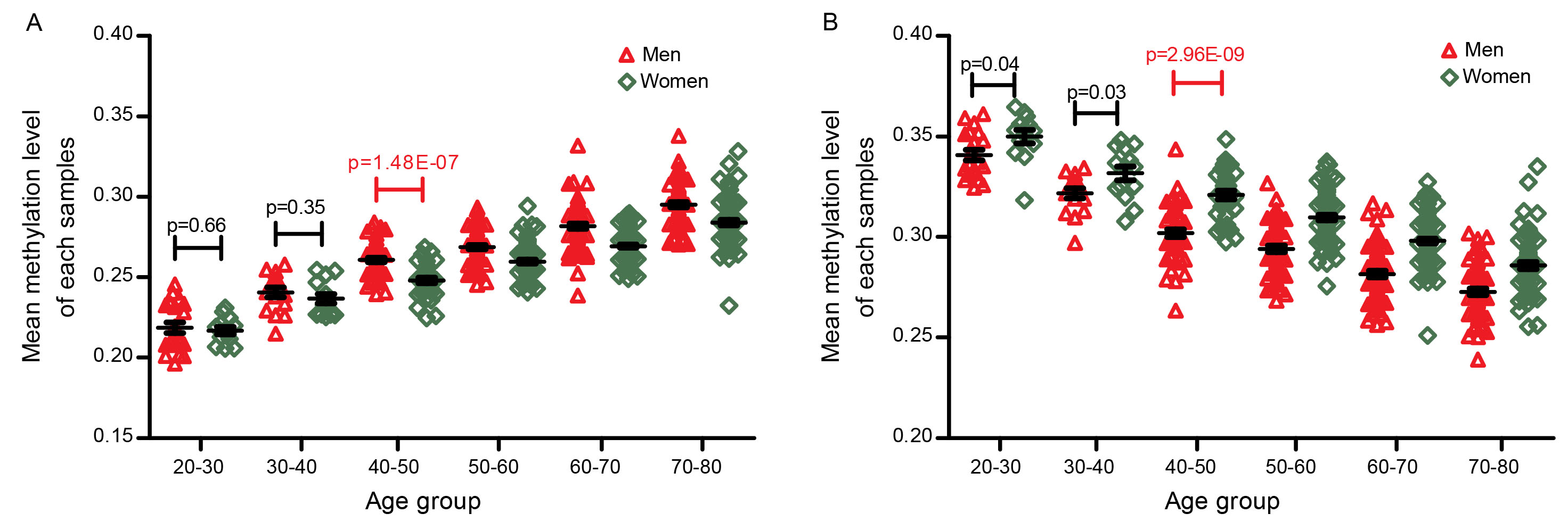


**Figure S2. Methylation differences of the identified 290 CpGs (with accelerated methylation changes in males) between the two sexes among different age groups.** (A) CpGs with positive associations between methylation level and age. (B) CpGs with negative associations between methylation level and age.


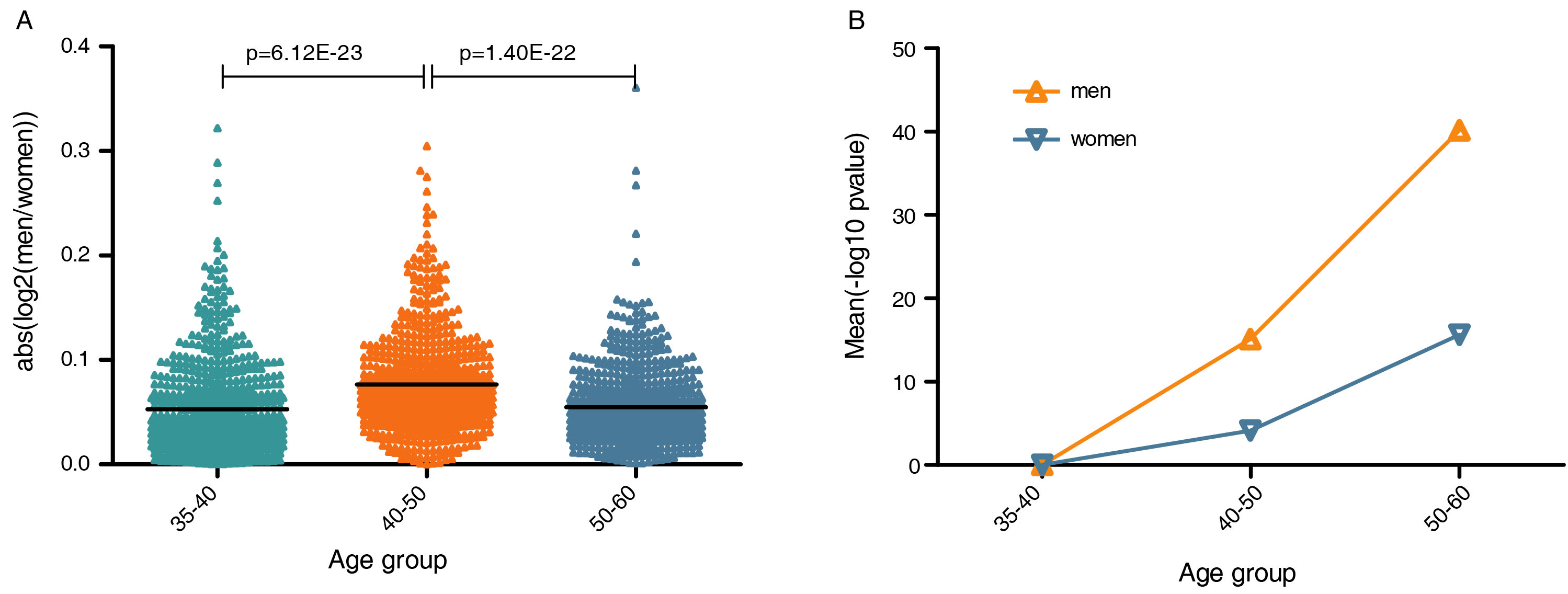


**Figure S3. Sexually dimorphic methylation in the CpGs with accelerated methylation changes in males.** (A) Methylation differences of the accelerated CpGs between the two sexes among different age groups. (B) Significant degree of methylation changes for the accelerated CpGs between older (e.g., 40–50 years old) and youngest (i.e., 35–40 years old) groups in each gender.

**Table S1.** Summary of the collected DNA methylation datasets.

|  | **GEO access number** | **Platform** | **Tissue** | **Women** | | | **Men** | | |
| --- | --- | --- | --- | --- | --- | --- | --- | --- | --- |
|  |  |  |  | **No.** | | **Age (years)** | | **No.** | **Age (years)** |
| 1 | GSE32148 | Illumina 450K array | blood | 362 | 5-101 | | 346 | | 3.5-96 |
|  | GSE41169 |  |  |  |  |  |  |  |  |
|  | GSE40279 |  |  |  |  |  |  |  |  |
| 2 | GSE55763 |  |  | 871 | 23.7-75 | | 1840 | | 24.5-74.8 |

**Table S2.** Information on the 290 accelerated CpG sites in the first dataset.

|  | qvalC_BH | extC_age_shift (years) | gene_name | Recoded in GeneCards V3.12 （√ represents "recoded"） | | | |
| --- | --- | --- | --- | --- | --- | --- | --- |
|  |  |  |  | cardiovascular disease | coronary heart disease | stroke | hypertention |
| cg00034468 | 1.93E-09 | -6.5904 | ACTA1 | × | × | × | × |
| cg00059225 | 2.87E-12 | -6.4483 | GLRA1 | × | × | × | × |
| cg00194146 | 7.66E-07 | -6.6002 | GFOD1 | × | × | × | × |
| cg00387658 | 1.01E-31 | -18.438 | CASS4 | × | × | × | × |
| cg00439658 | 1.43E-07 | -4.2039 | GRIN2C | × | × | × | × |
| cg00484358 | 1.75E-19 | -10.457 | ALX3 | × | × | √ | × |
| cg00503840 | 7.09E-07 | -5.5146 | DLX5 | √ | × | × | × |
| cg00602811 | 0.0046973 | -3.5473 | ZEB2 | √ | × | × | × |
| cg00664406 | 1.35E-24 | -15.069 | GRM2 | × | × | × | × |
| cg00695391 | 5.50E-26 | -15.448 | MMEL1 | √ | × | √ | √ |
| cg00741624 | 3.34E-11 | -6.8987 | UNC79 | × | × | × | × |
| cg00791074 | 0.007832 | -2.4217 | MTHFD1L | × | × | × | × |
| cg00985388 | 0.0015747 | -3.6511 | VWA5B2 | × | × | × | × |
| cg01034993 | 0.0004983 | -4.2063 | CORO2B | × | × | × | × |
| cg01101873 | 1.86E-24 | -15.121 | PRDM16 | × | × | × | × |
| cg01132064 | 0.0007684 | -3.6819 | DGKZ | × | × | × | × |
| cg01158574 | 5.47E-07 | -6.5981 | NTSR2 | × | × | × | × |
| cg01511567 | 1.12E-07 | -7.6272 | SSRP1 | × | × | × | √ |
| cg01777397 | 1.78E-09 | -8.5695 | CLIP4 | × | × | × | × |
| cg01812894 | 9.77E-08 | -7.4494 | ALDH1A1 | × | × | × | × |
| cg01856162 | 0.005104 | -4.2958 | ECEL1 | × | × | × | × |
| cg01889237 | 5.17E-12 | -7.7675 | OLIG3 | × | × | × | √ |
| cg02018902 | 3.46E-08 | -7.2304 | ANKRD34C | × | × | × | × |
| cg02046143 | 8.38E-07 | -5.4715 | IGSF9B | √ | × | × | × |
| cg02258201 | 3.54E-33 | -16.478 | HRCT1 | × | × | × | × |
| cg02342594 | 1.89E-06 | -6.8602 | LHX6 | × | × | × | × |
| cg02361903 | 1.17E-06 | -5.266 | CSF1 | × | × | × | × |
| cg02571816 | 4.99E-09 | -7.301 | PPP1R14A | × | × | × | × |
| cg03001305 | 0.0003367 | -4.1284 | STAT5A | × | × | × | × |
| cg03399905 | 1.80E-09 | -6.058 | ANKRD34C | × | × | √ | √ |
| cg03428981 | 0.0009884 | -3.1571 | EPHB6 | × | × | × | × |
| cg03484180 | 2.09E-10 | -8.4957 | MON1A | √ | √ | × | × |
| cg03545227 | 6.15E-06 | -6.2139 | PTPRN | √ | × | × | × |
| cg03555227 | 4.16E-06 | -4.8637 | RANBP17 | √ | √ | × | √ |
| cg03607117 | 1.08E-06 | -5.176 | SFMBT1 | × | × | × | × |
| cg04313338 | 4.45E-11 | -8.384 | SLC6A2 | × | × | × | × |
| cg04453050 | 7.45E-23 | -14.343 | GRM2 | √ | × | √ | × |
| cg04455430 | 1.84E-06 | -6.6159 | DMRTA2 | √ | × | × | × |
| cg04503319 | 6.05E-08 | -6.0927 | ANKRD11 | × | × | × | × |
| cg04528819 | 4.02E-08 | -8.1946 | KLF14 | × | × | × | × |
| cg04606861 | 3.01E-18 | -8.7098 | GALNT9 | × | × | × | × |
| cg04682133 | 8.55E-11 | -8.2531 | USP24 | √ | × | × | × |
| cg04858155 | 4.12E-05 | -5.5218 | ALX4 | √ | √ | × | √ |
| cg04865692 | 1.28E-07 | -6.3912 | KCNC3 | √ | √ | × | × |
| cg05110225 | 1.04E-05 | -6.4008 | SUMF2 | √ | √ | × | × |
| cg05266781 | 3.71E-05 | -4.5219 | IRX5 | × | × | × | × |
| cg05353133 | 4.75E-14 | -11.187 | ACTA1 | × | × | × | × |
| cg05492306 | 0.0015137 | -4.271 | ERCC1 | √ | × | × | × |
| cg05700079 | 4.52E-12 | -7.4258 | ZIC1 | × | × | × | × |
| cg06060874 | 3.40E-29 | -18.58 | PRDM16 | × | × | × | × |
| cg06069616 | 0.0010495 | -5.4894 | EVA1C | × | × | × | × |
| cg06335143 | 2.45E-08 | -5.7759 | ZYG11A | √ | × | × | √ |
| cg06400319 | 6.45E-13 | -10.083 | SPRR2B | × | × | × | × |
| cg06484360 | 0.0001786 | -5.1348 | LSM4 | √ | × | × | × |
| cg06493994 | 5.66E-10 | -6.6638 | SCGN | × | × | × | × |
| cg06602847 | 1.83E-12 | -6.7343 | BRINP1 | × | × | × | × |
| cg06618474 | 1.34E-10 | -9.997 | CRIP1 | √ | × | × | × |
| cg06707978 | 1.47E-09 | -6.3026 | ZIK1 | × | × | × | × |
| cg06737494 | 1.51E-06 | -6.8628 | GHSR | √ | √ | × | √ |
| cg06754224 | 5.11E-05 | -6.2308 | SESN2 | × | × | × | × |
| cg06911487 | 0.0004299 | -3.9454 | OLFM2 | × | × | × | × |
| cg06942685 | 9.18E-13 | -7.5417 | ZNF542P | × | × | × | × |
| cg06942701 | 3.73E-05 | -5.3118 | TBR1 | √ | √ | × | × |
| cg06969845 | 4.63E-13 | -11.524 | HRH2 | √ | × | × | √ |
| cg07127410 | 4.28E-13 | -10.252 | ZNRF3 | × | × | × | × |
| cg07211259 | 0.0026443 | -3.7536 | PDCD1LG2 | × | × | × | × |
| cg07502389 | 4.43E-11 | -6.2995 | NEFM | × | × | × | × |
| cg07539927 | 1.69E-06 | -7.0191 | CST6 | × | × | × | × |
| cg07892413 | 3.00E-06 | -5.9025 | HS6ST1 | √ | × | × | × |
| cg08081725 | 0.0001579 | -4.829 | NDE1 | √ | √ | √ | × |
| cg08231709 | 1.50E-10 | -9.3671 | KCNS2 | × | × | × | √ |
| cg08288130 | 0.0067207 | -3.7643 | DOK2 | × | × | × | × |
| cg08541518 | 1.50E-07 | -5.9296 | BAI3 | √ | × | × | × |
| cg08555657 | 6.42E-07 | -7.4328 | SPRR2E | × | × | × | × |
| cg08570034 | 4.47E-08 | -8.1321 | CPLX2 | × | × | × | × |
| cg08655844 | 2.01E-07 | -7.5882 | VOPP1 | × | × | × | × |
| cg09188763 | 0.0015475 | -4.0641 | LMO1 | √ | √ | × | × |
| cg09232937 | 2.74E-08 | -5.7937 | IRX1 | √ | × | × | × |
| cg09626984 | 5.28E-19 | -12.46 | GATA4 | × | × | × | × |
| cg09828265 | 7.75E-05 | -5.7366 | ITGA9 | × | × | × | × |
| cg10086328 | 2.00E-18 | -14.588 | PEX5 | √ | × | × | × |
| cg10290276 | 1.08E-06 | -6.2918 | ASCL2 | × | × | × | × |
| cg10308253 | 1.13E-05 | -7.2445 | ZC3H12D | × | × | × | × |
| cg10317026 | 0.002911 | -3.7736 | EFNA3 | × | × | × | × |
| cg10397932 | 9.45E-08 | -6.195 | SKI | × | × | × | √ |
| cg10406027 | 2.38E-05 | -5.5898 | ZC3H7A | √ | × | × | × |
| cg10806820 | 2.68E-14 | -8.1514 | CELSR3 | × | × | × | × |
| cg11120551 | 1.73E-11 | -11.227 | CHD1L | √ | × | × | × |
| cg11344352 | 0.0019193 | -2.6763 | ERCC1 | × | × | × | × |
| cg11367633 | 4.62E-05 | -5.389 | CHD6 | √ | × | × | √ |
| cg11700800 | 1.73E-08 | -6.2766 | NR2F1-AS1 | × | × | × | × |
| cg11705975 | 0.0014595 | -2.8693 | PRLHR | × | × | × | × |
| cg11890639 | 0.0003632 | -5.0964 | TEX264 | × | × | × | × |
| cg12052661 | 3.59E-07 | -5.4927 | CACNA1B | × | × | × | × |
| cg12103569 | 1.29E-16 | -10.746 | SFSWAP | × | × | × | × |
| cg12283460 | 4.58E-06 | -7.3178 | RNF111 | × | × | × | × |
| cg12339920 | 0.0092064 | -3.396 | TGFBI | × | × | × | × |
| cg12373771 | 2.97E-12 | -7.3413 | CECR6 | × | × | × | × |
| cg12419863 | 0.000149 | -4.7649 | PLAGL1 | × | × | × | × |
| cg12446246 | 3.91E-09 | -9.0517 | PLXNA2 | × | × | × | × |
| cg12589387 | 1.50E-09 | -10.072 | YTHDF1 | × | × | × | × |
| cg12626956 | 1.79E-10 | -8.1444 | FOXB2 | × | × | × | × |
| cg12878812 | 6.56E-15 | -7.7385 | SRRM4 | × | × | × | × |
| cg12934382 | 2.52E-13 | -7.4147 | GRM2 | × | × | × | × |
| cg13347071 | 6.91E-10 | -7.6418 | UNC80 | × | × | × | × |
| cg13381486 | 1.44E-10 | -8.3593 | AMER3 | × | × | × | × |
| cg13591783 | 0.0026539 | -3.9267 | ANXA1 | × | × | × | × |
| cg13593287 | 1.82E-08 | -7.5649 | SPTB | × | × | √ | √ |
| cg13790426 | 0.0055038 | -3.3549 | CLCC1 | × | × | × | × |
| cg13790576 | 3.94E-11 | -8.0109 | LCN6 | √ | √ | √ | √ |
| cg13868393 | 3.68E-06 | -7.406 | SEC14L1 | × | × | × | × |
| cg14042143 | 8.31E-11 | -8.3522 | IQCE | × | × | × | × |
| cg14068328 | 2.66E-12 | -10.425 | TBX18 | × | × | × | × |
| cg14113739 | 0.0044812 | -4.0272 | COL5A1 | × | × | × | × |
| cg14134497 | 2.49E-12 | -7.8433 | DTNA | × | × | × | × |
| cg14208102 | 0.0043541 | -3.1102 | TREX1 | √ | √ | × | √ |
| cg14244577 | 3.26E-08 | -6.6463 | DDX19B | × | × | × | × |
| cg14255824 | 4.23E-05 | -5.5304 | TJP2 | √ | × | × | √ |
| cg14361627 | 7.84E-12 | -6.2349 | KLF14 | × | × | × | × |
| cg14499385 | 1.39E-10 | -9.7048 | SLC40A1 | × | × | × | × |
| cg14517133 | 1.15E-08 | -8.4942 | PRPH2 | × | × | × | × |
| cg14519515 | 0.0003458 | -4.096 | ADRBK1 | × | × | × | × |
| cg14556683 | 0.0018502 | -2.6613 | EPHX3 | × | × | × | × |
| cg14837598 | 3.81E-19 | -11.26 | DMTN | × | × | × | × |
| cg14973055 | 5.17E-07 | -4.7374 | DNAI2 | × | × | × | × |
| cg15035705 | 0.0003652 | -4.5093 | MAML1 | × | √ | × | × |
| cg15037004 | 4.64E-05 | -6.7915 | ZNF366 | × | × | × | × |
| cg15149095 | 4.49E-08 | -8.8955 | SYT14 | × | × | × | × |
| cg15332241 | 0.000375 | -5.208 | MFSD5 | × | × | × | × |
| cg15480367 | 4.91E-07 | -4.9232 | CHGA | √ | × | × | × |
| cg15485728 | 4.32E-07 | -6.8658 | PRUNE2 | √ | √ | × | × |
| cg15736994 | 5.80E-12 | -8.8371 | ASIC5 | × | × | × | √ |
| cg15809217 | 0.0001882 | -5.3469 | BAG6 | × | × | × | × |
| cg16069986 | 3.53E-06 | -6.4247 | SHANK2 | × | × | × | √ |
| cg16171137 | 0.0002119 | -4.9877 | BAG6 | × | √ | × | × |
| cg16219603 | 5.16E-11 | -7.2174 | PENK | × | √ | × | × |
| cg16376108 | 1.19E-08 | -6.2267 | IRX5 | × | × | × | × |
| cg16686396 | 1.70E-30 | -18.446 | PRDM16 | × | × | × | × |
| cg16717122 | 3.00E-12 | -6.982 | SCG3 | √ | × | × | × |
| cg16785344 | 1.19E-05 | -5.1227 | PXN | × | × | × | × |
| cg16922810 | 5.06E-05 | -5.3822 | ARC | × | × | × | × |
| cg16928994 | 0.0098173 | -3.8058 | RBM45 | × | × | × | × |
| cg16983588 | 1.11E-22 | -14.637 | PRDM10 | × | × | × | × |
| cg16984944 | 7.11E-10 | -9.0602 | TBC1D23 | × | × | × | × |
| cg17024199 | 1.21E-08 | -8.4922 | MGAT5B | √ | √ | × | × |
| cg17163527 | 8.40E-08 | -8.1088 | MEGF6 | × | × | × | × |
| cg17247206 | 0.0066395 | -3.1032 | SNUPN | × | × | × | × |
| cg17280346 | 3.41E-10 | -6.7512 | ZIC1 | √ | √ | × | × |
| cg17306747 | 2.02E-08 | -6.8194 | SLC6A3 | × | × | × | × |
| cg17329534 | 0.0012369 | -3.653 | ZBTB7B | × | × | × | × |
| cg17436656 | 1.88E-08 | -5.9905 | RARG | × | × | × | × |
| cg17631451 | 0.0001056 | -4.2505 | TREX1 | × | × | × | × |
| cg18064714 | 1.44E-09 | -6.6028 | SP8 | × | × | √ | √ |
| cg18236477 | 7.62E-16 | -9.3441 | ATP8A2 | × | × | × | × |
| cg18338984 | 1.03E-07 | -6.5401 | IQCE | × | × | × | × |
| cg18473521 | 3.59E-18 | -10.268 | HOXC4 | × | × | × | × |
| cg18515624 | 1.32E-13 | -11.836 | GAREM | × | × | × | × |
| cg18738190 | 9.61E-09 | -6.1756 | CHST3 | × | × | × | × |
| cg19046959 | 1.74E-06 | -8.5053 | COL8A2 | × | × | × | × |
| cg19168338 | 0.0014674 | -4.4434 | CORO7 | √ | × | × | × |
| cg19407095 | 8.55E-11 | -8.7624 | SOX1 | × | × | × | × |
| cg19414741 | 2.78E-06 | -5.292 | PENK | √ | × | × | × |
| cg19416570 | 5.35E-12 | -10.624 | ZNF274 | × | × | × | × |
| cg19671120 | 2.86E-15 | -6.8626 | CNGA3 | × | × | × | × |
| cg19722847 | 9.12E-11 | -6.5944 | IPO8 | × | × | × | × |
| cg19761273 | 2.09E-09 | -6.701 | CSNK1D | × | × | × | × |
| cg19802138 | 5.02E-07 | -5.358 | SOX1 | × | × | × | × |
| cg19838043 | 4.88E-08 | -7.5937 | ZFYVE21 | × | × | × | × |
| cg19855470 | 6.60E-05 | -4.4319 | CACNA1I | √ | √ | × | √ |
| cg19891728 | 5.70E-10 | -6.6956 | ANK1 | √ | × | × | × |
| cg20068209 | 0.0003469 | -5.0006 | TMEM30A | × | × | × | × |
| cg20133890 | 0.0003153 | -4.3057 | LY6G6E | × | × | × | × |
| cg20143982 | 4.19E-10 | -9.0592 | PEX5 | × | × | × | × |
| cg20222376 | 4.88E-06 | -4.0755 | AKAP8L | √ | × | × | √ |
| cg20388732 | 1.35E-05 | -4.7082 | STAT5A | × | × | × | × |
| cg20813374 | 3.43E-06 | -5.4955 | FKBP5 | × | × | × | × |
| cg21255438 | 5.16E-11 | -9.0543 | PRDM14 | √ | × | × | × |
| cg21578195 | 3.34E-07 | -6.569 | PTK6 | √ | × | × | √ |
| cg21629821 | 0.0079067 | -4.0526 | DKFZp434J0226 | √ | × | × | × |
| cg21632975 | 5.36E-13 | -8.077 | NOVA2 | × | × | × | × |
| cg21644578 | 2.26E-05 | -5.9019 | ESPNP | × | × | × | × |
| cg21683284 | 2.88E-18 | -12.326 | DNAJC6 | √ | × | × | × |
| cg21709871 | 1.11E-14 | -11.366 | NRBP2 | × | × | × | × |
| cg22796704 | 9.87E-12 | -6.147 | ARHGAP22 | × | × | × | × |
| cg22820188 | 3.94E-11 | -10.534 | LMNA | × | × | × | × |
| cg23142799 | 2.45E-08 | -5.2046 | SHISA2 | × | × | × | × |
| cg23469878 | 2.15E-10 | -8.253 | LCN6 | × | × | × | × |
| cg23606718 | 1.63E-10 | -5.3958 | AMER3 | × | × | × | × |
| cg23753748 | 4.97E-10 | -7.7805 | CALHM2 | × | × | × | × |
| cg23760945 | 0.001191 | -4.6148 | ELOF1 | × | × | × | × |
| cg23998119 | 2.77E-12 | -10.607 | ZDHHC22 | × | √ | × | × |
| cg24199834 | 4.97E-10 | -7.5951 | POU4F2 | × | × | × | × |
| cg24466241 | 3.43E-11 | -5.6647 | ZYG11A | × | × | × | × |
| cg25447717 | 1.55E-05 | -6.0911 | VWA5B2 | √ | × | × | × |
| cg25468516 | 0.0007466 | -3.7045 | SIPA1 | √ | × | × | × |
| cg25478614 | 3.49E-07 | -5.9612 | SST | √ | × | × | × |
| cg25533247 | 6.46E-07 | -4.2683 | AKAP8L | × | × | × | × |
| cg25788793 | 3.18E-06 | -5.5933 | SLC2A9 | √ | × | √ | √ |
| cg25802093 | 8.50E-09 | -7.0289 | SPAG6 | × | × | × | × |
| cg26290632 | 1.51E-09 | -6.7219 | CALB1 | √ | × | √ | × |
| cg26316599 | 1.23E-11 | -8.6802 | ATP6V0E1 | × | × | × | × |
| cg26628907 | 0.001251 | -4.8441 | MED24 | × | × | × | × |
| cg26856607 | 1.63E-10 | -6.987 | NCAN | × | × | × | × |
| cg27187555 | 1.86E-24 | -18.402 | PRDM16 | √ | × | × | × |
| cg27209729 | 1.04E-05 | -5.3148 | NRXN2 | × | × | × | × |
| cg27213509 | 8.37E-08 | -6.5176 | EVX2 | × | √ | × | × |
| cg27320127 | 2.57E-12 | -5.6637 | KCNK12 | × | × | × | × |
| cg27530093 | 0.000175 | -5.2387 | CCDC97 | × | × | × | × |
| cg27544190 | 7.84E-08 | -6.7073 | EVA1C | × | × | × | × |
| cg00260802 | 3.39E-06 | -4.75 |  |  |  |  |  |
| cg00745389 | 0.0015183 | -3.8897 |  |  |  |  |  |
| cg00748589 | 5.33E-08 | -3.8781 |  |  |  |  |  |
| cg00875989 | 6.54E-07 | -5.1714 |  |  |  |  |  |
| cg01538166 | 3.38E-07 | -5.0264 |  |  |  |  |  |
| cg01770755 | 0.0002954 | -3.76 |  |  |  |  |  |
| cg01832712 | 0.0027552 | -4.1982 |  |  |  |  |  |
| cg01902066 | 1.12E-07 | -6.5387 |  |  |  |  |  |
| cg02129885 | 0.0001882 | -4.8096 |  |  |  |  |  |
| cg02318784 | 1.96E-08 | -5.7864 |  |  |  |  |  |
| cg02383785 | 0.001191 | -3.2636 |  |  |  |  |  |
| cg02492279 | 0.0035234 | -4.181 |  |  |  |  |  |
| cg02650266 | 4.67E-11 | -5.1351 |  |  |  |  |  |
| cg02843500 | 0.0001596 | -5.8327 |  |  |  |  |  |
| cg02991727 | 2.18E-11 | -9.233 |  |  |  |  |  |
| cg03443986 | 3.24E-09 | -5.6662 |  |  |  |  |  |
| cg03655330 | 0.0015969 | -4.1757 |  |  |  |  |  |
| cg04033732 | 0.0030181 | -3.7429 |  |  |  |  |  |
| cg04308040 | 1.99E-05 | -5.0825 |  |  |  |  |  |
| cg04434593 | 0.0054002 | -3.5634 |  |  |  |  |  |
| cg04578903 | 5.66E-12 | -7.1357 |  |  |  |  |  |
| cg04673912 | 4.43E-05 | -5.0494 |  |  |  |  |  |
| cg04751276 | 3.81E-08 | -7.4138 |  |  |  |  |  |
| cg05024939 | 3.25E-16 | -7.8801 |  |  |  |  |  |
| cg05207637 | 5.08E-12 | -8.6955 |  |  |  |  |  |
| cg05316627 | 2.74E-08 | -6.704 |  |  |  |  |  |
| cg05991454 | 1.53E-12 | -5.598 |  |  |  |  |  |
| cg06247837 | 2.77E-10 | -7.5737 |  |  |  |  |  |
| cg07027613 | 4.02E-08 | -5.3622 |  |  |  |  |  |
| cg07056299 | 1.51E-05 | -6.7054 |  |  |  |  |  |
| cg07851675 | 9.74E-08 | -7.117 |  |  |  |  |  |
| cg07871633 | 3.56E-08 | -6.1147 |  |  |  |  |  |
| cg08550421 | 2.91E-21 | -11.55 |  |  |  |  |  |
| cg09988805 | 0.0014377 | -4.7365 |  |  |  |  |  |
| cg10001186 | 0.0001041 | -3.8636 |  |  |  |  |  |
| cg10150592 | 2.60E-11 | -8.6935 |  |  |  |  |  |
| cg11323439 | 1.14E-09 | -7.7437 |  |  |  |  |  |
| cg11442608 | 9.50E-08 | -7.8719 |  |  |  |  |  |
| cg11524400 | 0.000449 | -4.4289 |  |  |  |  |  |
| cg11810998 | 0.0001375 | -6.4338 |  |  |  |  |  |
| cg13119578 | 0.0006254 | -3.7488 |  |  |  |  |  |
| cg13486406 | 4.14E-09 | -7.9408 |  |  |  |  |  |
| cg13649056 | 5.28E-08 | -5.0351 |  |  |  |  |  |
| cg13807549 | 1.38E-08 | -7.6311 |  |  |  |  |  |
| cg14314729 | 0.0002109 | -4.6295 |  |  |  |  |  |
| cg14469684 | 2.01E-17 | -12.953 |  |  |  |  |  |
| cg14585371 | 5.26E-08 | -4.9666 |  |  |  |  |  |
| cg15895690 | 3.48E-05 | -4.0849 |  |  |  |  |  |
| cg16541931 | 5.63E-14 | -8.476 |  |  |  |  |  |
| cg16932827 | 3.18E-11 | -6.7839 |  |  |  |  |  |
| cg17110586 | 3.79E-14 | -5.431 |  |  |  |  |  |
| cg17133388 | 5.54E-06 | -7.2963 |  |  |  |  |  |
| cg17214023 | 8.69E-05 | -5.4226 |  |  |  |  |  |
| cg17885226 | 8.80E-08 | -4.0562 |  |  |  |  |  |
| cg17939805 | 2.83E-08 | -5.2673 |  |  |  |  |  |
| cg18633600 | 6.57E-10 | -6.6221 |  |  |  |  |  |
| cg18660898 | 0.0004299 | -4.8117 |  |  |  |  |  |
| cg18826637 | 5.33E-08 | -7.8826 |  |  |  |  |  |
| cg18902090 | 2.85E-20 | -10.43 |  |  |  |  |  |
| cg18928900 | 4.23E-06 | -6.2391 |  |  |  |  |  |
| cg18933331 | 6.07E-24 | -9.1566 |  |  |  |  |  |
| cg19165390 | 7.94E-09 | -5.4684 |  |  |  |  |  |
| cg19356311 | 0.0025632 | -3.9697 |  |  |  |  |  |
| cg19663246 | 8.93E-11 | -9.0973 |  |  |  |  |  |
| cg19729744 | 5.08E-12 | -10.334 |  |  |  |  |  |
| cg20359994 | 7.70E-35 | -13.828 |  |  |  |  |  |
| cg20912205 | 8.29E-10 | -9.3222 |  |  |  |  |  |
| cg22013564 | 1.08E-06 | -6.4763 |  |  |  |  |  |
| cg22273555 | 6.45E-14 | -9.0649 |  |  |  |  |  |
| cg22358580 | 5.85E-15 | -11.795 |  |  |  |  |  |
| cg22682811 | 0.0014174 | -4.5534 |  |  |  |  |  |
| cg22737154 | 0.0035311 | -3.7976 |  |  |  |  |  |
| cg23190089 | 0.0032449 | -3.3927 |  |  |  |  |  |
| cg23500537 | 3.01E-18 | -7.1104 |  |  |  |  |  |
| cg25129541 | 3.51E-12 | -11.053 |  |  |  |  |  |
| cg26427498 | 0.0060409 | -3.4045 |  |  |  |  |  |
| cg26549701 | 5.60E-08 | -6.5951 |  |  |  |  |  |
| cg26557693 | 1.62E-06 | -5.7824 |  |  |  |  |  |
| cg26921969 | 8.20E-08 | -6.3982 |  |  |  |  |  |
| cg27215033 | 0.0001126 | -4.3927 |  |  |  |  |  |
| cg27536559 | 0.0045734 | -3.6031 |  |  |  |  |  |

**Table S3. Information on the 574 accelerated CpG sites in the second dataset.**

|  | qvalC_BH | estC_age_shift (years) | gene_name | Recoded in GeneCards V3.12 (√ represents "recorded") | | | |
| --- | --- | --- | --- | --- | --- | --- | --- |
|  |  |  |  | cardiovascular disease | coronary heart disease | stroke | hypertension |
| cg10247798 | 2.89E-13 | -5.8912 | ABHD14B | × | × | × | × |
| cg15736994 | 3.39E-07 | -3.8528 | ACCN5 | × | × | × | × |
| cg18770350 | 1.68E-06 | -3.9257 | ACTN2 | √ | × | × | × |
| cg26088662 | 4.01E-13 | -5.074 | ADRA1B | √ | √ | × | √ |
| cg20222376 | 2.33E-22 | -5.6606 | AKAP8L | × | × | × | × |
| cg25533247 | 1.19E-28 | -6.7038 | AKAP8L | × | × | × | × |
| cg02900766 | 5.13E-06 | -3.4519 | ALDH1A2 | √ | × | × | √ |
| cg00484358 | 3.55E-23 | -5.1366 | ALX3 | × | × | × | × |
| cg11071207 | 8.86E-13 | -5.5003 | ALX3 | × | × | × | × |
| cg04398950 | 5.01E-27 | -7.4316 | AMN | × | × | × | × |
| cg07435445 | 1.04E-08 | -4.6308 | AMN | × | × | × | × |
| cg19891728 | 7.75E-40 | -9.8019 | ANK1 | √ | × | × | × |
| cg01897498 | 4.53E-33 | -8.8973 | ANKRD19 | × | × | × | × |
| cg25316339 | 1.40E-06 | -2.6637 | ANKRD34B | × | × | × | × |
| cg02018902 | 1.41E-47 | -9.4846 | ANKRD34C | × | × | × | × |
| cg03399905 | 6.10E-22 | -5.4652 | ANKRD34C | × | × | × | × |
| cg04044664 | 0.00011653 | -2.7164 | ANKRD43 | × | × | × | × |
| cg02699218 | 5.28E-10 | -3.9798 | ANKRD43 | × | × | × | × |
| cg08415592 | 6.99E-06 | -2.7665 | APOL1 | × | √ | × | √ |
| cg22796704 | 6.83E-23 | -5.3352 | ARHGAP22 | × | × | × | × |
| cg24150153 | 9.64E-14 | -4.6022 | ARHGAP29 | × | × | × | × |
| cg10290276 | 2.50E-20 | -6.5498 | ASCL2 | √ | × | × | × |
| cg24856726 | 1.52E-05 | -3.4605 | ASCL4 | × | × | × | × |
| cg23368787 | 2.47E-31 | -8.7591 | ATP4A | × | × | × | × |
| cg18236477 | 7.04E-36 | -6.8304 | ATP8A2 | × | × | × | × |
| cg20382695 | 4.16E-13 | -5.2851 | ATRNL1 | √ | × | × | × |
| cg26727693 | 1.61E-17 | -7.0403 | AVPR1A | × | × | × | √ |
| cg10906284 | 2.96E-20 | -5.8993 | AVPR1A | × | × | × | √ |
| cg08541518 | 7.10E-30 | -6.6633 | BAI3 | √ | × | × | × |
| cg14131273 | 1.55E-12 | -5.487 | BARHL1 | × | × | × | × |
| cg17241310 | 1.41E-14 | -5.6272 | BARHL2 | × | × | × | × |
| cg15979173 | 7.14E-19 | -5.7103 | BARHL2 | × | × | × | × |
| cg18322569 | 4.02E-30 | -8.0662 | BARHL2 | × | × | × | × |
| cg14983606 | 1.25E-06 | -4.1801 | BHLHE22 | × | × | × | × |
| cg14043737 | 2.42E-22 | -6.7126 | BHLHE23 | × | × | × | × |
| cg03573446 | 4.69E-18 | -6.1517 | BMP7 | √ | × | √ | √ |
| cg03664992 | 4.33E-23 | -7.1518 | BMP8A | × | × | × | × |
| cg13806070 | 6.07E-06 | -3.3247 | BMP8A | × | × | × | × |
| cg11763509 | 4.54E-06 | -3.2535 | BMP8A | × | × | × | × |
| cg11139646 | 2.01E-05 | -3.2254 | BMP8B | × | × | × | × |
| cg04090392 | 1.47E-12 | -4.3404 | BNC1 | × | × | × | × |
| cg14385245 | 6.67E-16 | -5.2844 | BNC1 | × | × | × | × |
| cg17051321 | 7.73E-25 | -7.5826 | BNC1 | × | × | × | × |
| cg21117668 | 6.27E-06 | -2.4979 | BOK | × | × | × | × |
| cg21801378 | 8.68E-06 | -2.7025 | BRUNOL6 | × | × | × | × |
| cg20809087 | 2.13E-06 | -3.0019 | BRUNOL6 | × | × | × | × |
| cg24719321 | 8.52E-14 | -4.9551 | BSX | × | × | × | × |
| cg27314569 | 3.83E-17 | -5.9309 | BSX | × | × | × | × |
| cg10056132 | 3.40E-09 | -4.2311 | C10orf53 | × | × | × | × |
| cg24891133 | 5.93E-10 | -4.3936 | C13orf33 | × | × | × | × |
| cg01302656 | 1.87E-18 | -5.6967 | C14orf23 | × | × | × | × |
| cg12892303 | 1.68E-12 | -5.786 | C17orf104 | × | × | × | × |
| cg03643998 | 5.43E-06 | -2.9609 | C1QTNF1 | × | × | × | × |
| cg07027613 | 0.00087038 | -2.066 | C1RL | × | × | × | × |
| cg03509106 | 7.59E-07 | -4.1084 | C4orf31 | × | × | × | × |
| cg12052661 | 5.74E-31 | -7.5027 | CACNA1B | √ | × | √ | √ |
| cg11071401 | 2.16E-29 | -5.0059 | CACNA1G | √ | × | × | × |
| cg19855470 | 2.84E-06 | -3.1748 | CACNA1I | × | × | × | × |
| cg12451153 | 1.88E-18 | -5.8037 | CACNG2 | × | × | × | × |
| cg01666793 | 3.80E-06 | -3.5216 | CACNG8 | × | × | × | × |
| cg18335796 | 2.11E-17 | -6.6019 | CALB1 | × | × | × | √ |
| cg26290632 | 7.99E-20 | -4.3437 | CALB1 | × | × | × | √ |
| cg15108590 | 3.15E-08 | -4.0076 | CBS | √ | √ | √ | √ |
| cg22353329 | 2.06E-05 | -2.7283 | CBX4 | × | × | × | × |
| cg22108374 | 1.07E-24 | -6.1866 | CCDC33 | × | × | × | × |
| cg09688588 | 0.00792201 | -2.0265 | CCDC50 | × | × | × | × |
| cg04792813 | 2.27E-24 | -6.1789 | CCDC85C | × | × | × | × |
| cg24673101 | 1.61E-05 | -2.8019 | CCDC85C | × | × | × | × |
| cg12373771 | 1.29E-28 | -6.7242 | CECR6 | × | × | × | × |
| cg06268694 | 1.15E-10 | -4.5948 | CELSR1 | × | × | √ | √ |
| cg10806820 | 2.37E-71 | -9.8408 | CELSR3 | × | × | × | × |
| cg12422450 | 0.00033423 | -2.3625 | CHGA | √ | √ | × | √ |
| cg15480367 | 1.07E-13 | -3.6986 | CHGA | √ | √ | × | √ |
| cg16086620 | 1.03E-10 | -5.2491 | CHGA | √ | √ | × | √ |
| cg18738190 | 1.76E-49 | -10.506 | CHST3 | √ | × | × | × |
| cg04434593 | 1.39E-11 | -5.0526 | CLCF1 | × | × | × | × |
| cg01141812 | 9.50E-18 | -5.6437 | CLVS2 | × | × | × | × |
| cg19671120 | 2.46E-30 | -7.1742 | CNGA3 | × | × | × | √ |
| cg12920180 | 4.89E-06 | -3.0948 | COCH | × | × | × | × |
| cg18618815 | 6.06E-09 | -3.1137 | COL1A1 | √ | × | × | √ |
| cg23950157 | 2.46E-15 | -5.0162 | COL1A1 | √ | × | × | √ |
| cg27578811 | 6.56E-23 | -6.4494 | CPEB1 | × | × | × | × |
| cg09336320 | 1.31E-12 | -4.8991 | CPLX2 | × | × | √ | √ |
| cg23495748 | 9.92E-06 | -3.346 | CPLX2 | × | × | √ | √ |
| cg03544320 | 3.34E-05 | -3.2551 | CRMP1 | √ | × | × | × |
| cg19761273 | 1.31E-32 | -7.5568 | CSNK1D | × | × | × | × |
| cg21011139 | 1.86E-05 | -3.2725 | CYB5R2 | × | × | × | × |
| cg01410359 | 1.67E-14 | -4.8985 | CYP1B1 | √ | × | × | √ |
| cg02287710 | 6.26E-19 | -6.7718 | DIO3 | √ | × | × | × |
| cg15341124 | 7.57E-18 | -4.7289 | DIO3 | √ | × | × | × |
| cg00503840 | 4.25E-10 | -3.4699 | DLX5 | √ | × | × | × |
| cg04455430 | 9.13E-20 | -5.7561 | DMRTA2 | × | × | × | × |
| cg04931216 | 4.03E-11 | -5.2363 | DMRTA2 | × | × | × | × |
| cg14973055 | 7.38E-14 | -5.5153 | DNAI2 | × | × | × | × |
| cg26885220 | 4.09E-17 | -6.0655 | DNAJC6 | × | × | √ | √ |
| cg22458194 | 1.75E-13 | -5.1341 | DRD2 | √ | × | × | √ |
| cg14134497 | 4.63E-28 | -7.5635 | DTNA | √ | × | × | × |
| cg23413924 | 2.39E-16 | -5.3534 | DUOX1 | × | × | × | × |
| cg15824707 | 3.57E-18 | -6.3349 | DUOX2 | × | × | × | × |
| cg01135780 | 5.94E-12 | -4.9081 | EBF1 | √ | × | × | × |
| cg07525420 | 1.12E-07 | -3.2233 | EBF3 | √ | × | × | × |
| cg01739327 | 1.59E-08 | -4.3255 | EED | × | × | × | × |
| cg18582342 | 2.96E-16 | -5.9328 | ELAVL3 | √ | × | × | × |
| cg26153045 | 5.70E-16 | -5.6461 | ELN | √ | √ | × | √ |
| cg24724428 | 1.00E-09 | -1.9151 | ELOVL2 | × | × | × | × |
| cg14319235 | 1.03E-23 | -6.8298 | ELTD1 | √ | × | × | × |
| cg05825420 | 2.99E-15 | -5.6232 | EMX2OS | × | × | × | × |
| cg14556683 | 1.66E-18 | -3.8378 | EPHX3 | × | × | × | × |
| cg11344352 | 4.12E-23 | -5.9634 | ERCC1 | × | × | × | × |
| cg03335216 | 8.49E-07 | -3.9132 | ERRFI1 | √ | × | × | × |
| cg19560758 | 1.71E-19 | -4.8554 | ERRFI1 | √ | × | × | × |
| cg25755575 | 1.13E-08 | -3.9974 | ESRRG | × | × | × | × |
| cg27213509 | 3.26E-21 | -6.2553 | EVX2 | × | × | × | × |
| cg07920503 | 0.00013457 | -2.761 | FAM123A | × | × | × | × |
| cg13381486 | 2.83E-17 | -5.8943 | FAM123C | × | × | × | × |
| cg23606718 | 2.47E-53 | -6.5242 | FAM123C | × | × | × | × |
| cg13921352 | 1.02E-13 | -5.7827 | FAM19A4 | × | × | √ | √ |
| cg23967169 | 2.72E-09 | -4.2031 | FAM19A4 | × | × | √ | √ |
| cg13954457 | 4.79E-13 | -4.2016 | FBLL1 | × | × | × | × |
| cg26664161 | 2.95E-05 | -3.0644 | FBXL21 | × | × | × | × |
| cg05009601 | 1.36E-11 | -4.3709 | FEZF1 | × | × | × | × |
| cg11700800 | 2.69E-19 | -6.9578 | FLJ42709 | × | × | × | × |
| cg04501188 | 1.21E-05 | -3.1075 | FOXD2 | × | × | × | × |
| cg11848563 | 3.73E-22 | -6.781 | FOXD3 | √ | × | × | × |
| cg22815110 | 4.34E-20 | -6.6238 | FOXD3 | √ | × | × | × |
| cg18279094 | 3.44E-20 | -6.2317 | FOXD3 | √ | × | × | × |
| cg01281911 | 0.00118389 | -2.2034 | FOXE3 | × | × | × | × |
| cg03523785 | 1.86E-19 | -5.9639 | FOXG1 | √ | × | × | × |
| cg18299578 | 5.91E-15 | -5.6394 | FOXG1 | √ | × | × | × |
| cg07489048 | 3.25E-15 | -5.9767 | FOXG1 | √ | × | × | × |
| cg16038120 | 1.07E-10 | -4.1469 | FOXG1 | √ | × | × | × |
| cg25078444 | 0.00031061 | -2.3598 | FOXG1 | √ | × | × | × |
| cg23917057 | 3.93E-19 | -5.7388 | FOXI3 | × | × | × | × |
| cg20461912 | 1.86E-63 | -11.505 | FOXL2 | × | × | × | × |
| cg15829826 | 6.26E-14 | -6.0465 | FRMD8 | × | × | × | × |
| cg16498194 | 5.23E-14 | -5.9552 | FZD10 | × | × | × | × |
| cg20692569 | 2.75E-09 | -4.2572 | FZD9 | × | × | × | × |
| cg10676084 | 4.98E-20 | -6.8459 | GABRB3 | × | × | × | × |
| cg26447413 | 1.14E-06 | -3.9709 | GAS1 | √ | × | × | × |
| cg06704518 | 3.74E-35 | -8.0617 | GATA4 | √ | √ | √ | × |
| cg09626984 | 1.12E-59 | -11.68 | GATA4 | √ | √ | √ | × |
| cg14871932 | 8.08E-22 | -7.0741 | GCK | √ | √ | × | √ |
| cg25861699 | 8.08E-07 | -3.5955 | GCM2 | × | × | × | × |
| cg02328239 | 7.29E-15 | -4.7653 | GDNF | √ | × | √ | × |
| cg06737494 | 1.21E-30 | -5.6108 | GHSR | √ | √ | × | × |
| cg18920097 | 7.35E-11 | -4.4724 | GJD2 | √ | × | × | × |
| cg00059225 | 2.07E-57 | -7.6467 | GLRA1 | × | × | × | × |
| cg08316825 | 9.65E-13 | -5.6836 | GLRA1 | × | × | × | × |
| cg16541931 | 3.90E-50 | -8.4758 | GPR158 | × | × | × | × |
| cg25124276 | 1.93E-29 | -6.9921 | GPR158 | × | × | × | × |
| cg17497271 | 3.87E-09 | -3.8819 | GPR176 | × | × | × | × |
| cg15381304 | 5.30E-16 | -6.9779 | GPR6 | × | × | × | × |
| cg00613752 | 1.35E-14 | -5.9069 | GPR6 | × | × | × | × |
| cg10189695 | 1.33E-13 | -5.0203 | GPR78 | × | × | × | × |
| cg11970349 | 2.09E-07 | -2.3228 | GPR78 | × | × | × | × |
| cg21523251 | 3.18E-08 | -3.2571 | GPR78 | × | × | × | × |
| cg21296230 | 8.89E-06 | -2.3395 | GREM1 | × | × | × | √ |
| cg25148589 | 2.17E-16 | -6.1754 | GRIA2 | × | × | √ | × |
| cg23715749 | 1.38E-29 | -8.2443 | GRIK3 | × | × | × | × |
| cg00439658 | 3.20E-36 | -5.7098 | GRIN2C | × | × | × | × |
| cg07365960 | 1.85E-12 | -4.3963 | GRIN2C | × | × | × | × |
| cg04453050 | 3.98E-83 | -10.899 | GRM2 | × | × | × | × |
| cg12934382 | 4.84E-73 | -9.59 | GRM2 | × | × | × | × |
| cg00664406 | 1.53E-86 | -10.417 | GRM2 | × | × | × | × |
| cg15893346 | 1.03E-31 | -8.4062 | GUSB | × | × | √ | √ |
| cg07254032 | 1.38E-10 | -4.8686 | HCN1 | √ | × | × | × |
| cg18987410 | 0.00016574 | -2.652 | HEPACAM | × | × | × | × |
| cg18473521 | 2.99E-97 | -11.062 | HOXC4 | × | × | × | × |
| cg22358580 | 1.66E-51 | -8.6759 | HOXC4 | × | × | × | × |
| cg00495775 | 3.62E-25 | -7.1651 | HOXD11 | × | × | × | × |
| cg05942128 | 1.82E-31 | -7.5195 | HOXD11 | × | × | × | × |
| cg02390209 | 1.07E-25 | -9.0259 | HS3ST4 | × | × | × | × |
| cg18023598 | 3.49E-08 | -3.6947 | HTR6 | × | × | × | × |
| cg06638433 | 2.80E-07 | -3.7639 | IGF2BP1 | × | × | × | × |
| cg02046143 | 5.59E-13 | -5.3103 | IGSF9B | × | × | × | × |
| cg05213896 | 1.73E-06 | -2.309 | IL4I1 | × | × | × | × |
| cg09124496 | 2.59E-05 | -2.9008 | INHBA | √ | √ | × | √ |
| cg19284211 | 1.96E-11 | -4.7434 | INSM1 | × | × | × | × |
| cg13033938 | 8.65E-08 | -2.7909 | IP6K1 | × | × | × | × |
| cg19722847 | 5.10E-37 | -8.4979 | IPO8 | × | × | × | × |
| cg13702996 | 4.32E-22 | -6.6883 | IQSEC3 | × | × | × | × |
| cg00862117 | 1.32E-28 | -6.8734 | IQSEC3 | × | × | × | × |
| cg16851425 | 4.80E-08 | -3.83 | IRS2 | √ | √ | × | √ |
| cg09232937 | 6.93E-32 | -8.138 | IRX1 | √ | × | × | × |
| cg26578682 | 3.09E-28 | -7.7048 | IRX2 | × | × | × | × |
| cg05266781 | 1.20E-19 | -6.8107 | IRX5 | × | × | × | × |
| cg03927133 | 1.59E-27 | -7.0754 | ITPKA | × | × | × | × |
| cg26923490 | 1.15E-11 | -5.1671 | KCNA7 | × | × | × | × |
| cg18573383 | 1.40E-11 | -4.597 | KCNC2 | × | × | × | × |
| cg04865692 | 3.20E-40 | -9.3738 | KCNC3 | × | × | × | × |
| cg06572160 | 4.79E-21 | -6.1241 | KCNC3 | × | × | × | × |
| cg13902210 | 5.35E-15 | -4.5771 | KCNC4 | × | × | × | × |
| cg27320127 | 3.30E-51 | -7.6801 | KCNK12 | × | × | × | × |
| cg20491914 | 3.15E-10 | -5.4395 | KCNK3 | × | × | × | √ |
| cg08231709 | 8.30E-10 | -4.1749 | KCNS2 | × | × | × | × |
| cg01592801 | 3.70E-54 | -12.179 | KCNS2 | × | × | × | × |
| cg16832267 | 0.00062595 | -2.8314 | KCNS3 | × | × | × | × |
| cg23798387 | 9.24E-17 | -5.8454 | KIAA0100 | × | × | × | × |
| cg03391684 | 3.49E-12 | -5.0874 | KIAA1026 | × | × | × | × |
| cg15007156 | 1.51E-10 | -3.9081 | KIAA1026 | × | × | × | × |
| cg00741624 | 2.34E-22 | -6.0912 | KIAA1409 | × | × | × | × |
| cg15219685 | 6.32E-14 | -5.9707 | KIAA1919 | × | × | × | × |
| cg04528819 | 9.45E-20 | -4.6012 | KLF14 | × | × | × | × |
| cg08097417 | 5.29E-43 | -5.0531 | KLF14 | × | × | × | × |
| cg09499629 | 2.57E-30 | -5.2642 | KLF14 | × | × | × | × |
| cg14361627 | 7.91E-49 | -5.3137 | KLF14 | × | × | × | × |
| cg20426994 | 1.81E-09 | -4.6027 | KLF14 | × | × | × | × |
| cg00094518 | 2.38E-31 | -6.3129 | KLF14 | × | × | × | × |
| cg07955995 | 1.66E-25 | -4.6506 | KLF14 | × | × | × | × |
| cg22285878 | 6.56E-17 | -5.4957 | KLF14 | × | × | × | × |
| cg14507891 | 2.96E-05 | -2.805 | KLHDC8B | × | × | × | × |
| cg23718736 | 0.00035894 | -2.6353 | L3MBTL4 | × | × | × | × |
| cg19421125 | 3.09E-12 | -5.5698 | LAG3 | × | × | × | × |
| cg00237475 | 5.82E-19 | -6.0801 | LBXCOR1 | × | × | × | × |
| cg27470213 | 1.38E-12 | -4.4995 | LGALS3BP | √ | × | × | × |
| cg04061117 | 3.14E-06 | -3.5022 | LHFPL4 | × | × | × | × |
| cg20189674 | 9.42E-18 | -6.0571 | LHX1 | √ | × | × | × |
| cg20300246 | 1.97E-33 | -8.0614 | LHX3 | × | × | × | × |
| cg02342594 | 1.68E-22 | -6.7305 | LHX6 | × | × | × | × |
| cg00145253 | 2.19E-13 | -5.5067 | LHX8 | × | × | × | × |
| cg11842415 | 3.54E-06 | -3.6393 | LHX8 | × | × | × | × |
| cg12764034 | 5.43E-05 | -3.1343 | LHX8 | × | × | × | × |
| cg23049291 | 1.22E-18 | -6.7483 | LMX1A | × | × | × | √ |
| cg08138505 | 1.23E-05 | -3.0252 | LOC100130987 | × | × | × | × |
| cg13433250 | 4.43E-09 | -4.5391 | LOC100132215 | × | × | × | × |
| cg22197050 | 2.63E-06 | -3.2084 | LOC100132215 | × | × | × | × |
| cg00178249 | 2.62E-08 | -4.39 | LOC100192378 | × | × | × | × |
| cg10751726 | 9.46E-36 | -9.7709 | LOC150786 | × | × | × | × |
| cg17165841 | 4.17E-14 | -5.8515 | LOC200726 | × | × | × | × |
| cg09473315 | 8.96E-14 | -5.7991 | LOC283999 | × | × | × | × |
| cg18358723 | 0.00010749 | -2.6305 | LOC645323 | × | × | × | × |
| cg04604946 | 5.02E-13 | -6.4104 | LRRC23 | × | × | × | × |
| cg25044651 | 1.62E-12 | -5.4118 | LVRN | × | × | × | × |
| cg19211800 | 3.73E-08 | -4.2578 | MARCKS | × | × | × | × |
| cg16762684 | 3.61E-13 | -3.148 | MBP;MBP | × | × | × | × |
| cg13378934 | 5.87E-11 | -3.9845 | MEIS3 | × | × | × | × |
| cg04927004 | 4.54E-20 | -7.162 | MIR124-3 | × | × | × | × |
| cg07737781 | 5.13E-34 | -9.0431 | MLXIPL | √ | √ | √ | × |
| cg10092878 | 1.32E-32 | -9.3122 | MLXIPL | √ | √ | √ | × |
| cg13931228 | 2.21E-07 | -3.2537 | MPP6 | × | × | × | × |
| cg00791074 | 1.69E-51 | -9.8365 | MTHFD1L | × | √ | × | × |
| cg11359984 | 2.50E-09 | -4.7812 | MYLK | √ | √ | × | √ |
| cg00462994 | 5.96E-10 | -4.1775 | NAGS | × | × | × | × |
| cg03902729 | 6.91E-06 | -3.0005 | NAGS | × | × | × | × |
| cg00863306 | 1.71E-19 | -5.5827 | NANOS3 | × | × | × | × |
| cg16919569 | 1.76E-09 | -4.5282 | NBLA00301 | × | × | × | × |
| cg26856607 | 5.46E-25 | -7.3686 | NCAN | √ | √ | × | × |
| cg22234080 | 9.69E-20 | -6.9613 | NEFH | × | × | √ | × |
| cg07502389 | 1.01E-25 | -4.8497 | NEFM | √ | × | × | × |
| cg18267374 | 1.63E-16 | -4.5193 | NEFM | √ | × | × | × |
| cg07552803 | 9.14E-19 | -7.0789 | NEFM | √ | × | × | × |
| cg18898125 | 1.98E-05 | -2.3909 | NEFM | √ | × | × | × |
| cg03767531 | 2.17E-14 | -5.4383 | NEUROD1 | √ | × | × | × |
| cg19711579 | 4.52E-19 | -4.5467 | NEUROD1 | √ | × | × | × |
| cg17729667 | 1.00E-06 | -3.2828 | NINL | × | × | × | × |
| cg06874016 | 7.78E-08 | -3.8967 | NKIRAS2 | × | × | × | × |
| cg20049415 | 2.39E-11 | -5.1049 | NKX2-4 | × | × | × | × |
| cg21200656 | 4.65E-08 | -4.114 | NKX2-4 | × | × | × | × |
| cg11667020 | 4.12E-10 | -4.5005 | NKX2-4 | × | × | × | × |
| cg03968755 | 4.49E-27 | -6.9107 | NKX6-1 | × | × | × | × |
| cg21632975 | 1.19E-19 | -5.9823 | NOVA2 | × | × | × | × |
| cg18952796 | 3.62E-17 | -6.4415 | NPTX2 | × | × | × | × |
| cg05158615 | 9.78E-11 | -4.428 | NPY | √ | √ | √ | √ |
| cg01153166 | 1.34E-13 | -4.6474 | NR2F2 | √ | × | × | × |
| cg05470502 | 1.89E-07 | -3.9454 | NR5A2 | × | × | × | × |
| cg23091758 | 6.43E-05 | -2.2272 | NRIP3 | × | × | × | × |
| cg24711224 | 2.71E-28 | -8.5812 | NSD1;NSD1 | × | × | × | × |
| cg01449663 | 2.82E-16 | -6.024 | NTN1 | × | × | × | × |
| cg01158574 | 2.76E-23 | -6.912 | NTSR2 | × | × | × | × |
| cg09295081 | 3.71E-20 | -7.0192 | NTSR2 | × | × | × | × |
| cg17916490 | 1.52E-17 | -5.5803 | NTSR2 | × | × | × | × |
| cg15845821 | 4.00E-06 | -3.0253 | NWD1 | × | × | × | × |
| cg19784428 | 0.00501788 | -1.7381 | NWD1 | × | × | × | × |
| cg12597389 | 7.57E-18 | -5.7651 | NXPH1 | × | × | × | × |
| cg21567504 | 0.00132899 | -2.4298 | OCA2 | × | × | × | × |
| cg05207048 | 2.89E-06 | -2.909 | ODZ2 | × | × | × | × |
| cg06911487 | 4.14E-28 | -8.1294 | OLFM2 | × | × | × | × |
| cg01889237 | 3.25E-15 | -4.6493 | OLIG3 | √ | × | × | × |
| cg12744820 | 0.00011572 | -3.0646 | OLIG3 | √ | × | × | × |
| cg05215925 | 1.60E-27 | -7.7101 | OPRM1 | √ | √ | √ | × |
| cg12838303 | 3.69E-16 | -6.1906 | OPRM1 | √ | √ | √ | × |
| cg03957108 | 1.06E-11 | -4.5959 | OTX1 | × | × | × | × |
| cg08473330 | 8.31E-09 | -3.7198 | OTX1 | × | × | × | × |
| cg10658666 | 1.99E-07 | -3.7587 | OTX1 | × | × | × | × |
| cg03091551 | 2.64E-08 | -4.9813 | OTX2 | √ | × | × | × |
| cg06966811 | 2.90E-12 | -5.0091 | OTX2 | √ | × | × | × |
| cg23077820 | 3.13E-16 | -5.4701 | PAX3 | √ | × | × | × |
| cg23546474 | 7.73E-16 | -7.1126 | PAX3 | √ | × | × | × |
| cg01867395 | 4.51E-17 | -5.9685 | PAX6 | √ | √ | × | √ |
| cg13570972 | 7.71E-19 | -6.5834 | PAX6 | √ | √ | × | √ |
| cg07665387 | 3.13E-16 | -6.1664 | PCDH10 | × | × | × | × |
| cg05336395 | 3.93E-20 | -6.7685 | PCDH8 | × | × | × | × |
| cg08550421 | 2.88E-80 | -11.73 | PCDHA7 | × | × | × | × |
| cg16620537 | 1.81E-42 | -11.1 | PCDHA7 | × | × | × | × |
| cg18902090 | 6.33E-74 | -10.841 | PCDHA7 | × | × | × | × |
| cg23026864 | 1.17E-42 | -9.2975 | PCDHA7 | × | × | × | × |
| cg14566959 | 6.10E-07 | -3.088 | PCDHGA4 | × | × | × | × |
| cg15361590 | 6.53E-60 | -11.355 | PCDHGA4 | × | × | × | × |
| cg15672768 | 4.89E-12 | -5.7364 | PCDHGA4 | × | × | × | × |
| cg07211259 | 3.32E-09 | -3.4466 | PDCD1LG2 | √ | × | × | × |
| cg20119148 | 5.06E-21 | -5.8684 | PDE4C | × | × | × | × |
| cg17861230 | 5.55E-20 | -6.0766 | PDE4C | × | × | × | × |
| cg16219603 | 2.38E-18 | -5.087 | PENK | × | × | × | × |
| cg16419235 | 2.48E-15 | -3.5217 | PENK | × | × | × | × |
| cg10192893 | 3.00E-17 | -6.5411 | PHOX2B | √ | × | × | × |
| cg02110858 | 8.44E-37 | -11.206 | PLEC1 | × | × | × | × |
| cg22929506 | 7.89E-09 | -4.1921 | PNKD | × | × | × | × |
| cg18451114 | 5.63E-11 | -4.0251 | POU3F1 | × | × | × | × |
| cg03840594 | 2.10E-21 | -6.7247 | POU3F2 | × | × | × | × |
| cg27653384 | 7.34E-17 | -6.2324 | PPM1F | × | × | × | × |
| cg09515953 | 6.86E-28 | -7.3399 | PPP1R14A | × | × | × | √ |
| cg02571816 | 1.07E-30 | -7.1517 | PPP1R14A | × | × | × | √ |
| cg22557662 | 7.41E-21 | -6.6905 | PPP1R14A | × | × | × | √ |
| cg01295203 | 1.06E-18 | -7.0272 | PRDM14 | × | × | × | × |
| cg18035229 | 9.46E-24 | -6.87 | PRDM14 | × | × | × | × |
| cg21255438 | 7.08E-19 | -6.3035 | PRDM14 | × | × | × | × |
| cg00384539 | 1.04E-11 | -5.3958 | PRDM14 | × | × | × | × |
| cg13654588 | 2.78E-21 | -6.7459 | PRLHR | × | × | × | × |
| cg19392831 | 1.39E-09 | -3.8437 | PRLHR | × | × | × | × |
| cg11705975 | 3.05E-18 | -4.488 | PRLHR | × | × | × | × |
| cg08622677 | 2.19E-20 | -5.097 | PRMT8 | × | √ | × | × |
| cg02812207 | 5.35E-12 | -4.9036 | PRSS12 | × | × | × | × |
| cg15485728 | 5.36E-25 | -8.6776 | PRUNE2 | × | × | × | × |
| cg23804481 | 1.23E-23 | -6.0208 | PRUNE2 | × | × | × | × |
| cg03545227 | 1.75E-31 | -6.3357 | PTPRN | √ | × | × | × |
| cg12348202 | 6.43E-28 | -7.9219 | PTPRN2 | √ | × | √ | √ |
| cg22282410 | 8.25E-21 | -6.5891 | PTPRN2 | √ | × | √ | √ |
| cg11826475 | 7.74E-05 | -2.8819 | PXN;PXN | × | × | × | × |
| cg11480800 | 1.04E-14 | -5.7912 | PYGO1 | × | × | × | × |
| cg17841803 | 4.61E-13 | -5.4046 | PYGO1 | × | × | × | × |
| cg09676860 | 5.20E-47 | -11.265 | RAB6C | × | × | √ | √ |
| cg03002352 | 1.72E-15 | -4.8415 | RAD51AP2 | × | × | × | × |
| cg03555227 | 1.71E-24 | -6.9456 | RANBP17 | × | × | × | × |
| cg17436656 | 8.38E-22 | -6.4825 | RARG | √ | × | × | × |
| cg25309759 | 7.03E-12 | -5.3175 | RASL10B | × | × | × | × |
| cg17783401 | 2.28E-19 | -5.1199 | RGS20 | × | × | × | √ |
| cg10523019 | 2.96E-10 | -4.8616 | RHBDD1 | × | × | × | × |
| cg19451698 | 4.17E-13 | -5.3309 | RHBDD1 | × | × | × | × |
| cg01276475 | 1.20E-12 | -4.9819 | RIMS1 | × | × | × | × |
| cg07797372 | 7.88E-24 | -7.4608 | ROR1 | × | × | × | × |
| cg23488804 | 2.01E-10 | -4.2983 | RUNDC3A | × | × | × | × |
| cg12238343 | 6.36E-11 | -4.6238 | RXFP3 | × | × | × | × |
| cg03422911 | 3.58E-14 | -5.2657 | RYR2 | √ | × | × | × |
| cg05080154 | 1.37E-10 | -5.4591 | SALL3 | √ | × | × | × |
| cg03224418 | 8.27E-35 | -9.7695 | SAMD10 | × | × | × | × |
| cg26614073 | 0.00216246 | -2.3095 | SCAP | × | √ | × | × |
| cg16717122 | 4.76E-36 | -6.2996 | SCG3 | × | × | × | × |
| cg01230796 | 8.49E-25 | -6.4856 | SCGN | × | × | √ | × |
| cg06493994 | 2.21E-28 | -4.9767 | SCGN | × | × | √ | × |
| cg13029847 | 2.01E-10 | -4.4058 | SEZ6 | × | × | × | × |
| cg26307359 | 9.20E-16 | -6.1811 | SEZ6 | × | × | × | × |
| cg03607117 | 9.00E-06 | -2.0721 | SFMBT1 | × | × | × | √ |
| cg05476568 | 2.57E-15 | -6.3092 | SGEF | × | × | × | × |
| cg17977409 | 1.85E-06 | -3.5817 | SH3GL2 | √ | √ | × | × |
| cg07060551 | 4.04E-48 | -9.3162 | SHANK1 | × | × | × | × |
| cg00434010 | 1.86E-16 | -5.8923 | SIM1 | × | × | × | × |
| cg01850269 | 1.33E-15 | -4.33 | SIM1 | × | × | × | × |
| cg18528367 | 1.69E-09 | -4.424 | SIM1 | × | × | × | × |
| cg17380661 | 1.06E-05 | -3.8954 | SIM1 | × | × | × | × |
| cg14186066 | 5.78E-14 | -5.3019 | SIX6 | × | × | × | × |
| cg08209133 | 1.18E-06 | -3.4391 | SLC10A4 | × | × | × | × |
| cg07547549 | 2.57E-22 | -3.906 | SLC12A5 | × | × | × | × |
| cg02792538 | 0.00156579 | -2.3198 | SLC16A8 | × | × | × | × |
| cg04598517 | 2.78E-17 | -6.0556 | SLC6A3 | √ | × | × | √ |
| cg14692377 | 4.41E-10 | -2.8273 | SLC6A4 | √ | √ | √ | √ |
| cg23331484 | 5.66E-05 | -2.8899 | SLC7A10 | × | × | × | × |
| cg26890189 | 6.24E-26 | -6.805 | SLC8A2 | × | × | × | × |
| cg01236132 | 1.52E-12 | -4.7904 | SOX1 | × | × | × | × |
| cg04865691 | 1.05E-08 | -4.1506 | SOX1 | × | × | × | × |
| cg19407095 | 2.00E-41 | -9.5665 | SOX1 | × | × | × | × |
| cg16705627 | 2.05E-35 | -8.4658 | SOX1 | × | × | × | × |
| cg16330247 | 9.23E-20 | -7.0081 | SOX11 | √ | × | × | × |
| cg08044097 | 5.96E-20 | -6.6507 | SOX17 | √ | × | × | × |
| cg15377283 | 1.75E-13 | -5.9776 | SOX17 | √ | × | × | × |
| cg06825039 | 6.01E-14 | -6.1468 | SP8 | × | × | × | × |
| cg18064714 | 1.61E-19 | -4.492 | SP8 | × | × | × | × |
| cg01003961 | 2.10E-25 | -7.2125 | SP8 | × | × | × | × |
| cg18247055 | 4.92E-08 | -3.5621 | SPAG6 | × | × | × | × |
| cg23016129 | 6.04E-09 | -3.7391 | SPAG6 | × | × | × | × |
| cg12377139 | 5.37E-14 | -5.0659 | SPAG6 | × | × | × | × |
| cg12610471 | 7.43E-17 | -5.7471 | SPAG6 | × | × | × | × |
| cg13933080 | 2.36E-23 | -6.5439 | SPAG6 | × | × | × | × |
| cg02994974 | 3.53E-09 | -4.8554 | SPON1 | × | × | × | √ |
| cg05764628 | 9.79E-32 | -8.3613 | SPTBN4 | × | × | √ | √ |
| cg25237016 | 1.26E-27 | -7.9717 | SPTBN4 | × | × | √ | √ |
| cg12878812 | 2.76E-17 | -4.535 | SRRM4 | × | × | × | × |
| cg00481951 | 9.89E-32 | -4.8359 | SST | √ | √ | × | √ |
| cg05121480 | 1.71E-11 | -3.7578 | SST | √ | √ | × | √ |
| cg25478614 | 5.41E-33 | -5.7314 | SST | √ | √ | × | √ |
| cg17321954 | 5.37E-24 | -5.9495 | STXBP5L | × | × | × | × |
| cg10943458 | 9.83E-20 | -7.142 | STXBP5L | × | × | × | × |
| cg07806886 | 5.45E-17 | -4.4782 | STXBP5L | × | × | × | × |
| cg20591472 | 5.91E-20 | -4.8688 | SYPL2 | × | × | × | × |
| cg13673164 | 8.08E-07 | -3.8367 | SYPL2 | × | × | × | × |
| cg15149095 | 2.43E-22 | -6.883 | SYT14 | × | × | × | × |
| cg26158959 | 2.07E-24 | -6.7194 | SYT14 | × | × | × | × |
| cg19922137 | 0.00062731 | -2.8232 | SYT14 | × | × | × | × |
| cg12189835 | 2.27E-27 | -7.0309 | SYT7 | × | × | × | × |
| cg01287975 | 1.85E-08 | -3.4295 | TAC1 | √ | √ | √ | √ |
| cg17437939 | 0.00784347 | -2.1509 | TAC1 | √ | √ | √ | √ |
| cg05301866 | 5.78E-34 | -7.7761 | TBR1 | × | × | × | × |
| cg06488443 | 1.92E-18 | -6.3065 | TBR1 | × | × | × | × |
| cg12757011 | 3.14E-14 | -4.0856 | TBR1 | × | × | × | × |
| cg06942701 | 7.56E-27 | -7.9731 | TBR1 | × | × | × | × |
| cg02177231 | 3.83E-05 | -3.2012 | TBX15 | × | × | × | × |
| cg03989260 | 9.01E-12 | -4.8064 | TBX15 | × | × | × | × |
| cg23588217 | 4.52E-13 | -5.2526 | TBX15 | × | × | × | × |
| cg27262412 | 3.31E-08 | -3.7432 | TBX15 | × | × | × | × |
| cg09817427 | 9.02E-11 | -4.9608 | TBX18 | × | × | × | × |
| cg06247837 | 1.33E-27 | -7.8702 | TCAP | √ | × | × | √ |
| cg04467618 | 1.37E-15 | -4.8926 | TCF21 | √ | √ | × | × |
| cg04692403 | 4.33E-11 | -5.1753 | TCF21 | √ | √ | × | × |
| cg04940570 | 1.25E-16 | -4.4738 | TEAD1 | √ | × | × | × |
| cg01542019 | 1.74E-07 | -4.5663 | TECR | × | × | × | × |
| cg23244289 | 4.38E-05 | -3.272 | THBS4 | √ | √ | × | × |
| cg02248826 | 5.66E-28 | -7.9692 | TJP1 | √ | √ | × | × |
| cg14255824 | 3.04E-43 | -8.1457 | TJP2 | × | × | × | × |
| cg03998104 | 2.10E-07 | -4.0642 | TLX2 | × | × | × | × |
| cg25942450 | 1.13E-19 | -6.5368 | TLX3 | × | × | × | × |
| cg26844246 | 2.13E-19 | -6.8011 | TLX3 | × | × | × | × |
| cg00590036 | 3.96E-27 | -8.3179 | TMEM181 | × | × | × | × |
| cg19593767 | 1.33E-09 | -4.9751 | TOX2 | √ | × | × | × |
| cg22348673 | 1.09E-12 | -5.3138 | TRIL | × | × | × | × |
| cg24853724 | 5.02E-05 | -1.872 | TRIL | × | × | × | × |
| cg06448705 | 3.84E-10 | -4.4355 | TRPC7 | × | × | × | × |
| cg04084157 | 9.88E-35 | -7.091 | VGF | × | × | × | × |
| cg21186299 | 2.19E-28 | -5.7653 | VGF | × | × | × | × |
| cg02898293 | 1.95E-18 | -6.3707 | VSX1 | × | × | × | × |
| cg08739433 | 4.42E-10 | -3.6298 | WNT2B | × | × | × | × |
| cg09388605 | 1.41E-17 | -6.7212 | XKR4 | × | × | × | × |
| cg02662828 | 2.95E-06 | -3.0039 | ZAR1 | × | × | × | × |
| cg17953764 | 0.0001149 | -2.3648 | ZAR1 | × | × | × | × |
| cg08578136 | 5.66E-18 | -6.1682 | ZDBF2 | × | × | × | × |
| cg22162281 | 0.00067064 | -2.7377 | ZDBF2 | × | × | × | × |
| cg23998119 | 3.92E-49 | -9.7564 | ZDHHC22 | × | × | × | × |
| cg13786089 | 2.90E-12 | -5.1592 | ZFR2 | × | × | × | × |
| cg05371578 | 4.73E-25 | -6.3425 | ZIC1 | √ | × | × | × |
| cg05700079 | 9.01E-12 | -4.067 | ZIC1 | √ | × | × | × |
| cg16181396 | 1.87E-09 | -2.9272 | ZIC1 | √ | × | × | × |
| cg23448486 | 1.33E-18 | -6.6215 | ZIC1 | √ | × | × | × |
| cg04738965 | 1.16E-08 | -4.1024 | ZIC1;ZIC1 | × | × | × | × |
| cg17882660 | 6.75E-16 | -6.3031 | ZIC2 | √ | × | × | × |
| cg06707978 | 2.33E-13 | -4.4809 | ZIK1 | × | × | × | × |
| cg21911021 | 1.32E-07 | -3.3086 | ZIK1 | × | × | × | × |
| cg11294513 | 4.73E-19 | -6.6841 | ZNF154 | × | × | × | × |
| cg05661282 | 2.00E-12 | -5.6346 | ZNF154 | × | × | × | × |
| cg07788092 | 6.29E-09 | -4.9534 | ZNF177 | × | × | × | × |
| cg25334393 | 1.31E-05 | -2.8091 | ZNF251 | × | × | × | × |
| cg06942685 | 2.70E-08 | -3.3217 | ZNF542 | × | × | × | × |
| cg06458239 | 1.44E-22 | -6.3577 | ZNF549 | × | × | × | × |
| cg10635122 | 1.55E-14 | -5.5023 | ZNF577 | × | × | × | × |
| cg00008033 | 2.64E-15 | -5.0007 | ZNF613 | × | × | × | × |
| cg07568841 | 0.00036268 | -2.8855 | ZNRF2 | × | × | × | × |
| cg06335143 | 2.28E-12 | -3.7152 | ZYG11A | × | × | × | × |
| cg18184411 | 8.82E-07 | -3.1006 | ZYG11A | × | × | × | × |
| cg24466241 | 1.67E-10 | -3.2911 | ZYG11A | × | × | × | × |
| cg06784991 | 9.90E-18 | -3.799 | ZYG11A | × | × | × | × |
| cg16015712 | 1.07E-10 | -3.3896 | ZYG11A | × | × | × | × |
| cg00116092 | 9.60E-06 | -2.8718 |  |  |  |  |  |
| cg00949753 | 2.08E-10 | -4.7003 |  |  |  |  |  |
| cg01352586 | 3.76E-11 | -4.5415 |  |  |  |  |  |
| cg04103761 | 7.03E-12 | -4.4974 |  |  |  |  |  |
| cg06533314 | 7.86E-11 | -4.9542 |  |  |  |  |  |
| cg08023265 | 1.72E-12 | -5.2326 |  |  |  |  |  |
| cg13927566 | 2.97E-28 | -7.3248 |  |  |  |  |  |
| cg18343474 | 4.86E-38 | -7.0317 |  |  |  |  |  |
| cg18933331 | 1.09E-96 | -11.918 |  |  |  |  |  |
| cg23605843 | 2.47E-09 | -3.9474 |  |  |  |  |  |
| cg00073837 | 4.24E-09 | -4.3168 |  |  |  |  |  |
| cg00393837 | 5.83E-10 | -4.2607 |  |  |  |  |  |
| cg03443986 | 2.27E-11 | -4.0965 |  |  |  |  |  |
| cg06567855 | 1.30E-08 | -3.7071 |  |  |  |  |  |
| cg14674720 | 4.53E-33 | -5.8772 |  |  |  |  |  |
| cg14912644 | 7.17E-05 | -2.7434 |  |  |  |  |  |
| cg18826637 | 2.82E-44 | -8.9899 |  |  |  |  |  |
| cg18969232 | 2.92E-11 | -5.431 |  |  |  |  |  |
| cg19049194 | 3.44E-09 | -4.1677 |  |  |  |  |  |
| cg22303418 | 8.88E-11 | -4.6091 |  |  |  |  |  |
| cg25385733 | 4.29E-23 | -7.1751 |  |  |  |  |  |
| cg27215033 | 2.34E-14 | -5.4803 |  |  |  |  |  |
| ch.2.207814544R | 0.00010833 | -2.4458 |  |  |  |  |  |
| cg02232208 | 7.54E-32 | -8.993 |  |  |  |  |  |
| cg08541155 | 3.39E-10 | -4.3795 |  |  |  |  |  |
| cg10506882 | 1.36E-11 | -4.5062 |  |  |  |  |  |
| cg10796805 | 2.88E-05 | -2.9483 |  |  |  |  |  |
| cg15504461 | 0.00020778 | -2.7769 |  |  |  |  |  |
| cg18143296 | 0.00015933 | -2.4437 |  |  |  |  |  |
| cg00481350 | 1.12E-17 | -5.293 |  |  |  |  |  |
| cg05024939 | 2.56E-19 | -5.4102 |  |  |  |  |  |
| cg05106770 | 0.0001124 | -2.827 |  |  |  |  |  |
| cg05350411 | 4.23E-26 | -8.1302 |  |  |  |  |  |
| cg06711175 | 5.10E-06 | -3.1633 |  |  |  |  |  |
| cg07851675 | 3.39E-41 | -7.3588 |  |  |  |  |  |
| cg18032969 | 4.67E-22 | -7.713 |  |  |  |  |  |
| cg21166964 | 5.35E-15 | -5.5153 |  |  |  |  |  |
| cg22551065 | 3.15E-14 | -5.4602 |  |  |  |  |  |
| cg23500537 | 3.11E-30 | -4.0214 |  |  |  |  |  |
| cg23713079 | 1.74E-17 | -7.1603 |  |  |  |  |  |
| cg26549701 | 2.11E-29 | -8.3039 |  |  |  |  |  |
| cg26921969 | 7.33E-37 | -5.749 |  |  |  |  |  |
| cg03074925 | 1.06E-09 | -4.3823 |  |  |  |  |  |
| cg05316627 | 1.21E-06 | -3.1039 |  |  |  |  |  |
| cg17885226 | 1.43E-27 | -5.4108 |  |  |  |  |  |
| cg18928900 | 5.94E-16 | -6.7048 |  |  |  |  |  |
| cg20359994 | 1.85E-80 | -14.282 |  |  |  |  |  |
| cg21860429 | 3.25E-14 | -5.4875 |  |  |  |  |  |
| cg24698655 | 2.39E-05 | -2.4964 |  |  |  |  |  |
| ch.6.33611621F | 0.00287524 | -1.6033 |  |  |  |  |  |
| cg00745389 | 5.16E-23 | -5.7428 |  |  |  |  |  |
| cg02383785 | 7.03E-24 | -5.0043 |  |  |  |  |  |
| cg04427498 | 3.75E-10 | -4.0745 |  |  |  |  |  |
| cg13486406 | 1.56E-16 | -5.8882 |  |  |  |  |  |
| cg26557693 | 5.80E-17 | -6.073 |  |  |  |  |  |
| cg05236677 | 1.59E-17 | -6.6746 |  |  |  |  |  |
| cg18026631 | 1.43E-06 | -3.11 |  |  |  |  |  |
| cg00959431 | 3.29E-16 | -5.3569 |  |  |  |  |  |
| cg03262554 | 1.42E-23 | -6.0828 |  |  |  |  |  |
| cg13649056 | 2.03E-33 | -5.8253 |  |  |  |  |  |
| cg13823169 | 1.05E-25 | -6.4209 |  |  |  |  |  |
| cg23553442 | 6.43E-18 | -6.2982 |  |  |  |  |  |
| cg25427880 | 1.63E-07 | -2.9514 |  |  |  |  |  |
| cg05045027 | 8.84E-09 | -4.2955 |  |  |  |  |  |
| cg07178006 | 1.25E-17 | -5.6023 |  |  |  |  |  |
| cg10001186 | 2.05E-16 | -6.3977 |  |  |  |  |  |
| cg13119578 | 8.64E-07 | -3.1354 |  |  |  |  |  |
| cg14934413 | 4.10E-14 | -4.6432 |  |  |  |  |  |
| cg19389884 | 1.33E-09 | -4.1692 |  |  |  |  |  |
| cg00748589 | 1.84E-16 | -3.939 |  |  |  |  |  |
| cg03810428 | 2.39E-19 | -6.9492 |  |  |  |  |  |
| cg10778288 | 9.45E-20 | -4.8322 |  |  |  |  |  |
| cg24611446 | 8.26E-17 | -6.1189 |  |  |  |  |  |
| cg12852499 | 2.83E-16 | -6.1258 |  |  |  |  |  |
| cg23361092 | 5.10E-05 | -3.3074 |  |  |  |  |  |
| ch.13.39564907R | 0.00020943 | -1.9344 |  |  |  |  |  |
| cg02360199 | 1.58E-10 | -4.6035 |  |  |  |  |  |
| cg06821999 | 2.34E-14 | -4.3196 |  |  |  |  |  |
| cg23815900 | 2.00E-12 | -5.4821 |  |  |  |  |  |
| cg24035598 | 5.54E-08 | -3.9072 |  |  |  |  |  |
| cg27050153 | 1.71E-26 | -7.8473 |  |  |  |  |  |
| ch.14.97331099F | 0.00050354 | -2.0522 |  |  |  |  |  |
| cg01770755 | 8.41E-20 | -6.1769 |  |  |  |  |  |
| cg02983424 | 5.97E-05 | -2.927 |  |  |  |  |  |
| cg04751276 | 4.06E-14 | -5.2084 |  |  |  |  |  |
| cg12856183 | 2.12E-09 | -4.5057 |  |  |  |  |  |
| cg00101260 | 9.96E-08 | -3.9322 |  |  |  |  |  |
| cg01461824 | 3.08E-10 | -4.8927 |  |  |  |  |  |
| cg03431918 | 3.86E-14 | -4.2835 |  |  |  |  |  |
| cg04169021 | 9.03E-14 | -5.0301 |  |  |  |  |  |
| cg15001747 | 3.91E-08 | -3.6567 |  |  |  |  |  |
| cg15557036 | 1.61E-05 | -2.52 |  |  |  |  |  |
| cg00875989 | 3.59E-19 | -5.9377 |  |  |  |  |  |
| cg01352175 | 2.45E-15 | -6.1614 |  |  |  |  |  |
| cg08076830 | 3.92E-08 | -4.1893 |  |  |  |  |  |
| cg16061498 | 2.59E-22 | -6.3812 |  |  |  |  |  |
| cg17939805 | 8.64E-20 | -6.5126 |  |  |  |  |  |
| cg17110586 | 3.63E-34 | -4.8378 |  |  |  |  |  |
| cg21725716 | 8.64E-07 | -3.8351 |  |  |  |  |  |
| cg00260802 | 1.35E-07 | -3.9291 |  |  |  |  |  |
| cg17737621 | 3.33E-14 | -5.5599 |  |  |  |  |  |
| cg22682811 | 0.00193413 | -1.7915 |  |  |  |  |  |
| cg04753583 | 3.03E-12 | -4.5365 |  |  |  |  |  |
| cg07639287 | 1.87E-07 | -3.4932 |  |  |  |  |  |
| cg17326555 | 1.85E-06 | -3.438 |  |  |  |  |  |
| cg19306368 | 2.72E-09 | -3.6071 |  |  |  |  |  |
| cg14001664 | 9.62E-30 | -7.859 |  |  |  |  |  |
| cg16361302 | 2.01E-05 | -3.4097 |  |  |  |  |  |
| cg02650266 | 1.03E-37 | -5.9465 |  |  |  |  |  |
| cg05991454 | 5.09E-16 | -5.0535 |  |  |  |  |  |
| cg12765028 | 1.31E-40 | -9.8508 |  |  |  |  |  |
| cg16512661 | 2.89E-18 | -5.9945 |  |  |  |  |  |
| cg12172057 | 0.00054687 | -2.5997 |  |  |  |  |  |
| cg02352240 | 9.07E-07 | -3.4456 |  |  |  |  |  |
| cg07149609 | 2.47E-20 | -7.3483 |  |  |  |  |  |
| cg06782035 | 5.83E-25 | -4.9019 |  |  |  |  |  |
| cg09017434 | 1.12E-19 | -6.1799 |  |  |  |  |  |
| cg10616795 | 0.00389079 | -1.835 |  |  |  |  |  |
| cg15168727 | 6.11E-34 | -7.8643 |  |  |  |  |  |
| cg22995692 | 1.65E-15 | -6.2716 |  |  |  |  |  |
| cg23479922 | 2.36E-23 | -6.0292 |  |  |  |  |  |
| cg03738025 | 2.83E-16 | -5.383 |  |  |  |  |  |
| cg08445802 | 5.95E-11 | -4.686 |  |  |  |  |  |
| cg17066943 | 3.71E-26 | -6.897 |  |  |  |  |  |
| cg20442599 | 8.71E-29 | -6.498 |  |  |  |  |  |
| cg23746497 | 1.54E-12 | -4.8484 |  |  |  |  |  |
| cg00552235 | 1.38E-18 | -7.1699 |  |  |  |  |  |
| cg18057109 | 3.24E-20 | -6.8681 |  |  |  |  |  |
| cg18961681 | 4.92E-21 | -6.7445 |  |  |  |  |  |
| cg14353137 | 1.67E-11 | -5.4753 |  |  |  |  |  |
| cg15427886 | 3.44E-14 | -5.5329 |  |  |  |  |  |
| cg17694795 | 9.08E-09 | -4.2717 |  |  |  |  |  |
| cg24935332 | 1.15E-15 | -6.3046 |  |  |  |  |  |
| cg12225226 | 1.79E-08 | -3.7151 |  |  |  |  |  |
| cg24221648 | 8.30E-09 | -5.1708 |  |  |  |  |  |
| cg19464804 | 4.41E-18 | -7.066 |  |  |  |  |  |
| cg07336350 | 2.90E-17 | -4.4951 |  |  |  |  |  |
| cg10320659 | 8.44E-24 | -5.8844 |  |  |  |  |  |
